# STIM-IP3R crosstalk regulates migration of breast cancer cells

**DOI:** 10.1083/jcb.202411203

**Published:** 2025-07-28

**Authors:** Ruslana Militsin, Hadas Achildiev Cohen, Maya Hershfinkel, Ofek Levi, Stavit Drori, Adi Yifat Raz, Yuval Shaked, Raz Palty

**Affiliations:** 1 Department of Biochemistry, Technion Integrated Cancer Center, Ruth and Bruce Rappaport Faculty of Medicine, Technion - Israel Institute of Technology, Haifa, Israel; 2 Department of Cell Biology and Cancer Science, Technion Integrated Cancer Center, Ruth and Bruce Rappaport Faculty of Medicine, Technion Israel Institute of Technology, Haifa, Israel

## Abstract

Calcium ions (Ca^2+^) are crucial second messengers involved in numerous processes including tumorigenesis and cancer cell migration. Previous studies have shown that the endoplasmic reticulum (ER) Ca^2+^ sensors, stromal interaction molecules STIM1 and STIM2, are key regulators of cancer cell migration. In this study, using breast cancer cells lacking one or both STIM isoforms we show that although STIM proteins are critical regulators of cell migration, they are dispensable for this cellular activity. The mechanism underlying this complex effect involves functional crosstalk between STIM proteins and inositol 1,4,5-trisphosphate receptors (IP3Rs). Our findings indicate that beyond their classical role in store-operated Ca^2+^ entry, STIM proteins shape the spatial dynamics of IP3R-mediated Ca^2+^ release. Our results suggest that following ER Ca^2+^ depletion, the activated STIM proteins shift the pattern of IP3R-mediated Ca^2+^ release from a localized signal, which promotes cell migration, to a more diffuse signal, which attenuates cell migration.

## Teaser

STIM1 and STIM2 are critical regulators of breast cancer cell migration. Militsin et al. reveal novel crosstalk between STIM proteins and IP3Rs, whereby STIM activation alters the spatial pattern of IP3R-mediated Ca^2+^ release, shifting it from a promigratory localized signal to a diffuse, migration-suppressing signal.

## Introduction

Breast cancer is the most prevalent form of cancer in women, with metastasis remaining the main cause of death (Sung et al., 2021). Most types of breast cancer cells arise from mesenchymal transformation of epithelial cells, and the metastatic progression of these cells hinges on their capability to migrate from the initial tumor site toward blood or lymph vessels or to nearby tissues (Sarrió et al., 2008). Mesenchymal cell migration is a complex process involving various steps including cycles of formation and retraction of membrane protrusions at the cell leading edge followed by focal adhesion formation, generation of contractile forces along the cell axis, and disassembly of focal adhesion at the retracting cell tail (SenGupta et al., 2021). Remarkably, highlighting the importance of the cellular Ca^2+^ signaling machinery in mesenchymal cell migration, all of these processes are regulated by changes in the levels of cytosolic calcium (Tsai and Meyer, 2012; Wei et al., 2009; Yang et al., 2009; Iamshanova et al., 2017). Intracellular calcium signals are generated primarily via Ca^2+^ entry and Ca^2+^ release channels that are localized to the cell membrane or to membranes of intracellular Ca^2+^ stores, respectively. The inositol 1,4,5-trisphosphate receptor (IP3R) Ca^2+^ release channel and the store-operated Ca^2+^ entry (SOCE) channel are ubiquitously expressed and functionally linked Ca^2+^ shuttling mechanisms that have been shown to play important roles in the migration of breast cancer cells (Mound et al., 2017; Yang et al., 2009). During cell migration, a variety of physical and chemical cues stimulate phospholipase C–coupled cell surface receptors that generate the diffusible second messenger 1,4,5-trisphosphate (IP3), which in turn bind to and activate the IP3R on the membrane of the endoplasmic reticulum (ER). There are three IP3R isoforms (ITPR1/2/3). Although each isoform possesses distinct biophysical properties, the activation of either isoform leads to the mobilization of ER Ca^2+^ via two spatially and temporally distinct mechanisms: transient Ca^2+^ release events (Ca^2+^ puffs) from IP3R clusters and persistent Ca^2+^ release from a spatially diffuse pool of IP3Rs (Lock and Parker, 2020). Recent studies have shown that the KRAP protein is crucial for both localized and diffuse modes of IP3R-mediated Ca^2+^ release (Thillaiappan et al., 2021; Vorontsova et al., 2022). However, the mechanism of action and identity of regulatory proteins governing the transition between these Ca^2+^ release modes remains poorly understood. Opening of IP3Rs decreases luminal ER Ca^2+^ levels, which in turn trigger permeation of Ca^2+^ across the plasma membrane (PM) via SOCE. Calcium entry via SOCE allows refilling of the ER and generates both localized and global cellular Ca^2+^ signals. SOCE is primarily mediated by two key components: the ER-localized Ca^2+^ sensing protein stromal interaction molecule (STIM) and the PM-localized pore-forming subunit Orai protein (Liou et al., 2005; Roos et al., 2005; Zhang et al., 2005; Feske et al., 2006; Peinelt et al., 2006; Prakriya et al., 2006; Yeromin et al., 2006). A decrease in ER Ca^2+^ levels liberates Ca^2+^ from a luminal-facing EF-hand Ca^2+^ binding domain of STIM causing extensive conformational changes in its luminal and cytosolic facing regions. In the Ca^2+^-unbound active conformation, STIM interacts initially with PM polyphosphoinositides and clusters at ER-PM junctions where it then traps and activates Orai channels to initiate Ca^2+^ entry into the cell (Liou et al., 2005; Park et al., 2009; Yuan et al., 2009; Maléth et al., 2014). STIM and IP3R exhibit multiple modes of reciprocal regulation. By liberating Ca^2+^ from the ER, the IP3R contributes to activation of STIM, and in some cells, it has been shown to possess an additional function, distinct from its Ca^2+^-releasing capacity, whereby when ligated to IP3 it amplifies the association between STIM1 and Orai1 at the ER-PM junction (Chakraborty et al., 2023). Likewise, by facilitating the refilling of Ca^2+^ in the ER the STIM proteins enable sustained ER Ca^2+^ release via IP3Rs and they too mediate an additional regulatory function, independent from their Orai channel binding–activation capacity, whereby they have been shown to both positively (Béliveau et al., 2014) and negatively (Emrich et al., 2021) regulate Ca^2+^ release via IP3Rs. The molecular basis of this intricate regulatory mechanism is poorly understood, and whether it is important for mesenchymal cell migration is unknown. Here, we investigated the impact of alterations in Ca^2+^ signaling through SOCE and IP3R on the migration of breast cancer cells. We show that while deletion of either STIM1 or STIM2 inhibits the migration of cancer cells, deletion of both STIM isoforms (dKO) does not. The STIM-deficient cells display characteristics of mesenchymal cell migration; however, their migratory behavior becomes hypersensitive to changes in IP3R activity. Mechanistically, we find that following ER Ca^2+^ depletion STIM proteins facilitate a mode of diffuse Ca^2+^ release via IP3Rs. Our results indicate that STIM proteins play a crucial role not only in regulation of SOCE but also in maintaining a delicate equilibrium between localized Ca^2+^ puff and diffuse modes of calcium release through IP3Rs. The interplay between STIMs and IP3Rs is critical for mesenchymal cell migration.

## Results

### STIM proteins are essential for breast cancer cell seeding

**Figure S1.**
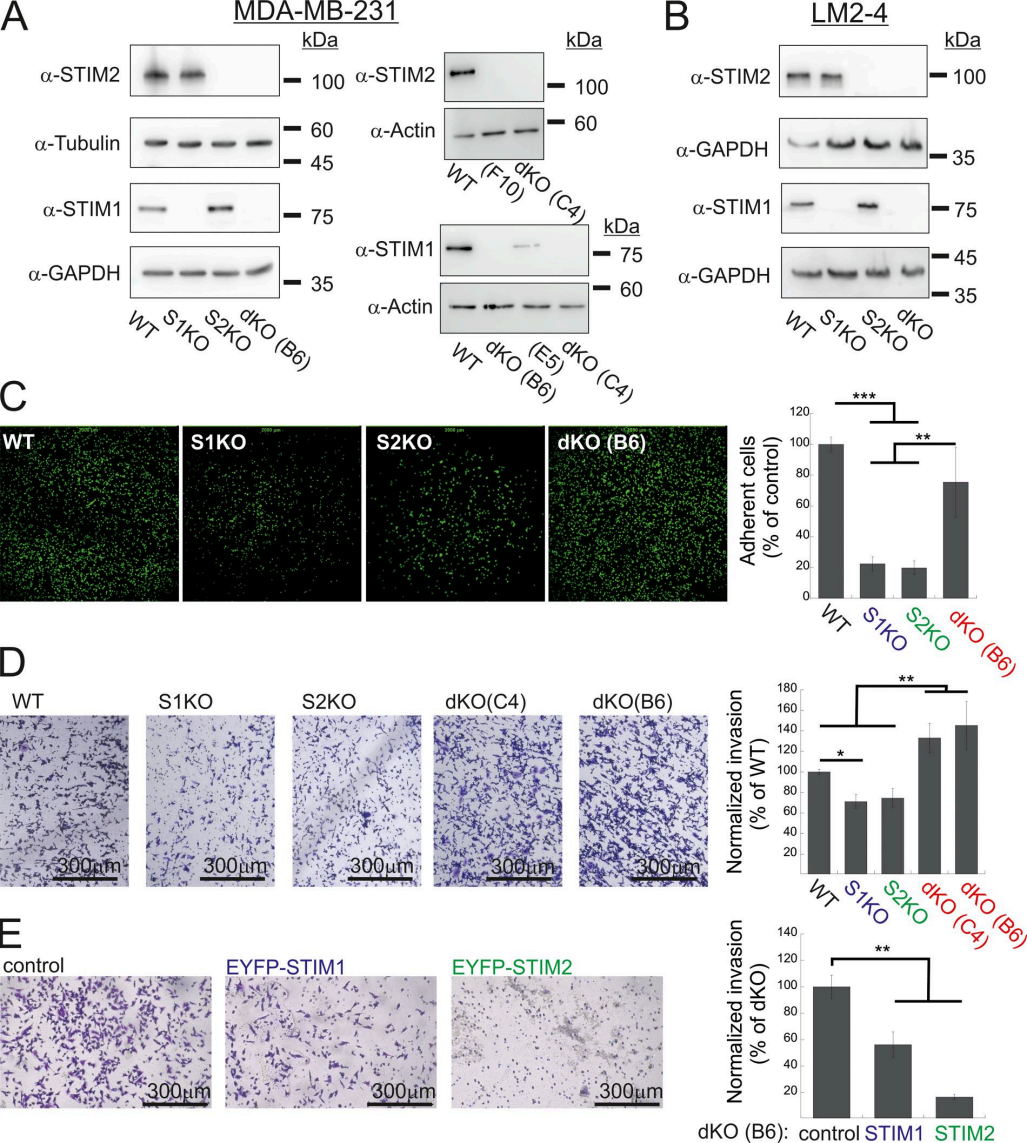
**Additional data related to** Fig. 1 **. (A and B)** Expression of STIM1 and STIM2 in MDA-MB-231 (A) and LM2-4 (B) cells following CRISPR/Cas9 gene editing. Representative images from WB analysis of lysates prepared from the indicated MDA-MB-231 (A) or LM2-4 (B) cells using antibodies for STIM1, STIM2, actin, GAPDH, or tubulin. Clones F10 and E5 are shown here as additional controls and were not used in the present study. **(C)** Representative images (left) and quantitation (right) of adhesion assays (*n* = 5) using the indicated MDA-MB-231 cells. The field of view in each image measures 2 × 2 mm. **(D and E)** Representative images (left) and quantitation (right) of two invasion assays using WT, S1KO, S2KO, dKO (C4), or dKO(B6) MDA-MB-231 cells (D) or using dKO(B6) cells re-expressing EYFP-STIM1 or EYFP-STIM2 (E). Scale bar = 300 μm. Results from each experiment in C or in D were normalized to WT cells, while data from E were normalized to control dKO cells. Bars show the mean ± SEM. Statistics: one-way ANOVA with Tukey’s post hoc test; *P < 0.05, **P < 0.01, ***P < 0.001. Source data are available for this figure: SourceData FS1.

**Figure 1.**
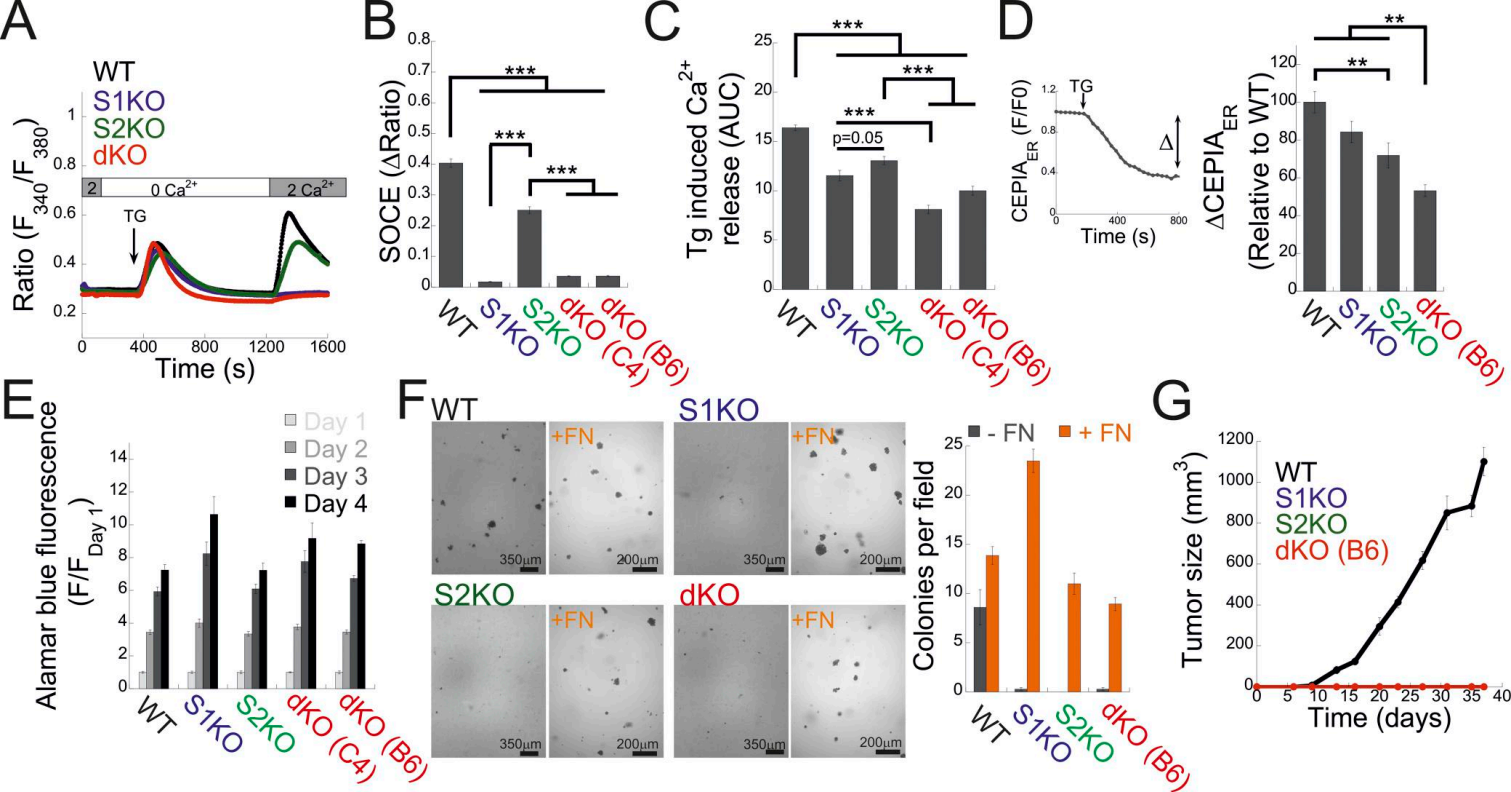
**STIM proteins are essential for breast cancer cell seeding in a soft extracellular environment. (A)** Average intracellular Ca^2+^ responses of SOCE are shown for WT (black, *n* = 126), S1KO (blue, *n* = 77), S2KO (green, *n* = 124) or for two independent clones of S1/S2 dKO (red; trace shows average of the two clones, B6, *n* = 46; C4, *n* = 54 cells). **(B and C)** Quantification (bars show the mean ± SEM) of SOCE (B) and Tg-induced ER Ca^2+^ release (C) for individual cells shown in (A). AUC is the area under the curve from time point 400 to 1,200 s. **(D)** ER Ca^2+^ was measured by tracking the fluorescence of G-CEPIA following treatment with Tg. Right inset shows a representative trace from WT cell illustrating the Tg-induced change in ER Ca^2+^ (Δ). Left, bars show the mean ± SEM of normalized ΔER Ca^2+^ for WT (*n* = 57), S1KO (*n* = 45), S2KO (*n* = 31), or S1/S2 dKO (B6, *n* = 42) cells. **(E)** Normalized alamarBlue absorbance (bars show the mean ± SEM, *n* = 3) at the indicated days following cell seeding is shown for WT, S1KO, S2KO, or dKO cells. **(F)** Left, representative images of colonies of the indicated type of cells formed on soft agar with or without embedded fibronectin (FN, orange) after 3 wk. Right, quantification of the average number of individual colonies per field of view, as indicated. Bars show the mean ± SEM from two experiments. The scale bar is 350 or 200 μm, as indicated. **(G)** Time course of average tumor growth after cancer cell inoculation is shown for the indicated cell types (five or six mice in each group). Note that no tumors were detected in mice injected with S1KO, S2KO, or dKO cells. Statistics: one-way ANOVA with Tukey’s post hoc test; *P < 0.05, **P < 0.01, ***P < 0.001.

To investigate the function of STIM1 and STIM2 proteins during metastatic progression, we utilized CRISPR/Cas9 gene editing to create multiple clones of MDA-MB-231 cells deficient in either STIM1 (S1KO), STIM2 (S2KO), or both isoforms (dKO) (Fig. S1 A). Consistent with earlier reports (Emrich et al., 2021), deletion of STIM2 mildly decreased SOCE, while deletion of STIM1 alone or together with STIM2 abolished SOCE (Fig. 1, A and B). The average levels of Ca^2+^ liberated from the ER by the SERCA inhibitor thapsigargin (Tg), used as an estimate of resting ER luminal Ca^2+^ levels, were higher in WT cells compared with all STIM-deficient cells (Fig. 1 C). In line with these results, analysis of ER Ca^2+^ content using an ER-targeted Ca^2+^ indicator (G-CEPIA_ER_) showed that resting ER Ca^2+^ levels were lower in STIM dKO cells compared with all other cell groups (Fig. 1 D). Notably, both analyses also detected a decrease in ER Ca^2+^ levels in S1KO or S2KO cells as compared to WT cells. Deletion of either STIM isoform did not affect cell proliferation or formation of colonies on soft agar supplemented with fibronectin (Fig. 1, E and F). However, in contrast to WT cells, STIM-deficient cells failed to form colonies when grown on soft agar lacking fibronectin (Fig. 1 F) and failed to form tumors when injected to the mammary fat pad of NOD/SCID mice (Fig. 1 G). This indicates a crucial role of either STIM isoform in the initial stages of breast cancer cell seeding.

### STIM proteins are dispensable for cell migration yet play a significant regulatory role in this process

**Figure 2.**
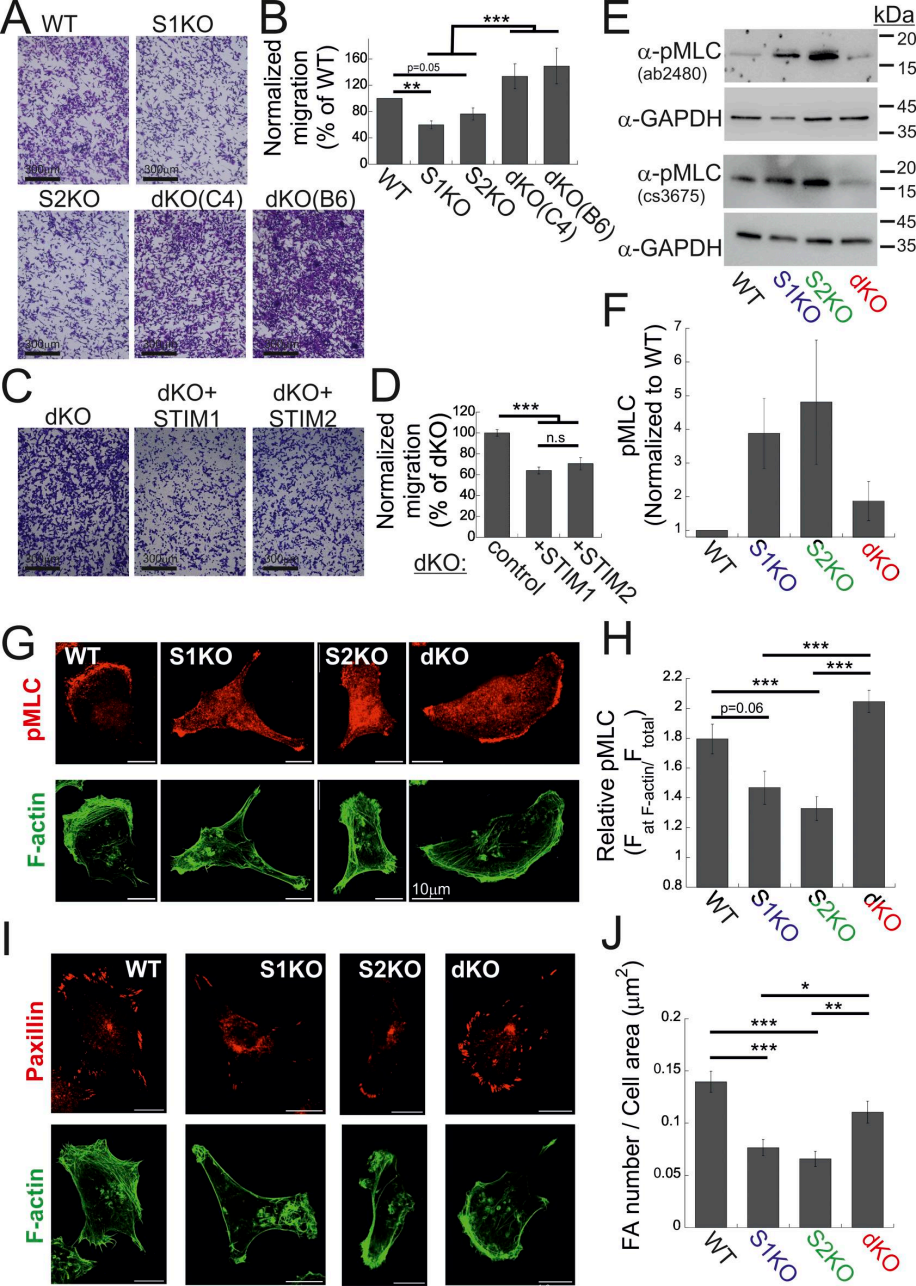
**Deletion of both STIM isoforms rescues cell migration. (A)** Representative images of WT, S1KO, S2KO, or dKO migrating cells, as indicated. **(B)** Scale bar = 350 μm (B)Number of migrating cells from each group was normalized to that of control (WT) cells. Bars show the mean ± SEM (WT, *n* = 18; S1KO, *n* = 13; S2KO, *n* = 15; dKO-C4, *n* = 8; dKO-B6, *n* = 7). **(C and D)** EYFP-STIM1 or EYFP-STIM2 was re-expressed in dKO cells. **(C)** Representative images of migrating cells from the indicated cells. Scale bar = 350 μm. **(D)** Number of migrating cells from STIM1 or STIM2 rescue cells was normalized to that of control (dKO) cells. Bars show the mean ± SEM (control, *n* = 7; STIM1, *n* = 6; STIM2, *n* = 7). **(E and F)** WB analysis of lysates from WT, S1KO, S2KO, and dKO cells using two different antibodies against pMLC (ab2480 or cs3675) and GAPDH as a loading control. **(E)** Representative images from two independent experiments. **(F)** Quantification of pMLC signal intensity relative to that of GAPDH and normalized to control (WT) cells. **(G and H)** Bars shows the mean ± SEM of normalized pMLC (*n* = 4) (G and H). Representative confocal images (G) and quantification (H) of anti-phosphorylated MLC (red) and phalloidin (F-actin, green) fluorescence (see the Materials and methods section). Bars show the mean ± SEM of pMLC staining at actin bundles per cell in the indicated cell type (WT, *n* = 30; S1KO, *n* = 28; S2KO, *n* = 32; dKO, *n* = 30). **(I and J)** Representative images (I) and quantification (J) of anti-paxillin (red) and phalloidin (F-actin, green) fluorescence (see the Materials and methods section). Bars show the mean ± SEM of FAs per cell in the indicated cell type (WT, *n* = 30; S1KO, *n* = 34; S2KO, *n* = 31; dKO, *n* = 36). Scale bar = 10 μm. Statistics: one-way ANOVA with Tukey’s post hoc test; *P < 0.05, **P < 0.01, ***P < 0.001. Source data are available for this figure: SourceData F2.

**Figure S2.**
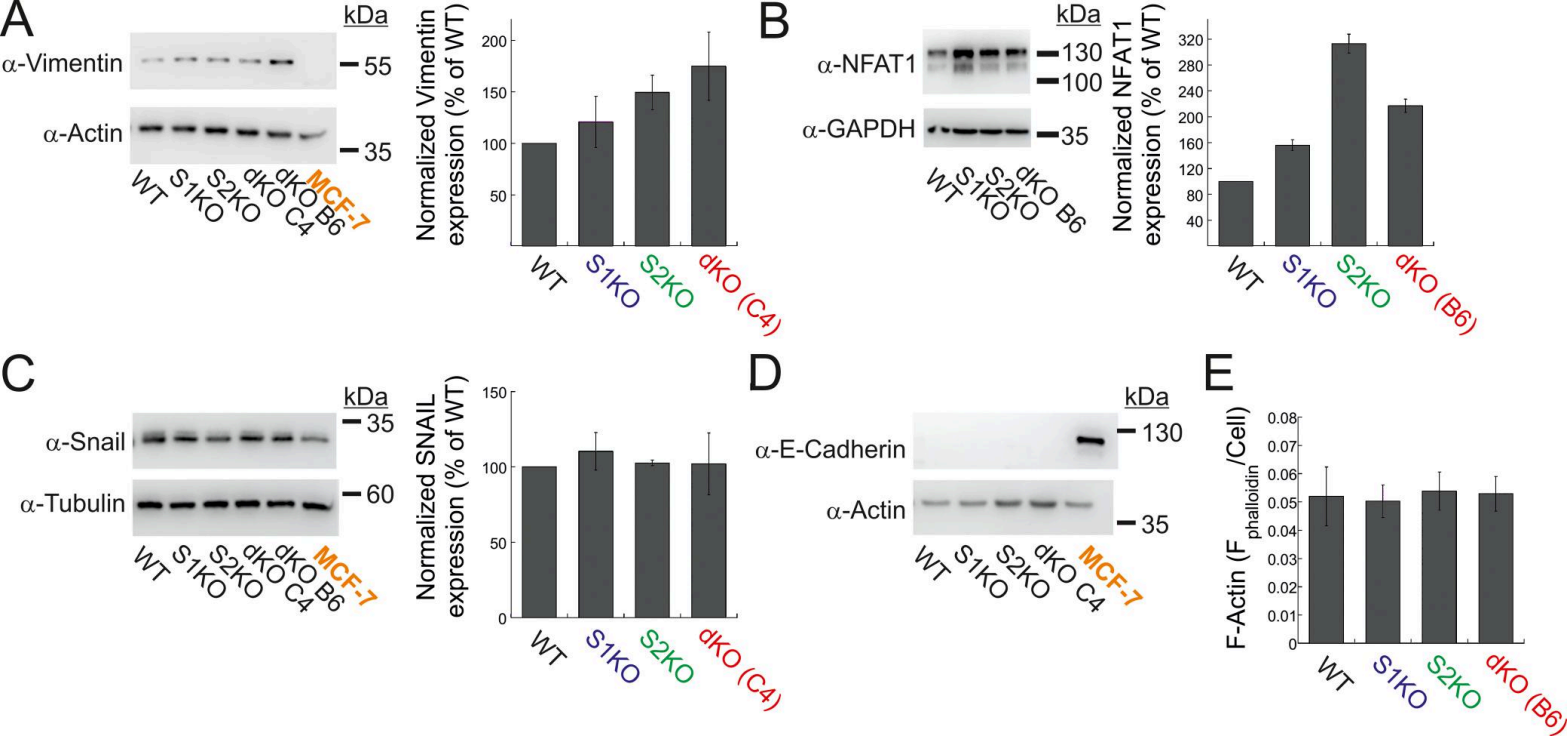
**Expression of EMT markers and NFAT1 in MDA-MB-231 and MCF-7 cells. (A–D)** Representative images (left) and quantitation (right) of WB analysis (*n* = 3–4) of lysates prepared from the indicated MDA-MB-231 or MCF-7 cells using antibodies for vimentin and actin (A), NFAT1 and GAPDH (B), Snail and tubulin (C), or E-cadherin and actin (D). **(E)** Quantification of total F-actin content in the indicated MDA-MB-231 cells (see Materials and methods). Bars show the mean ± SEM. Source data are available for this figure: SourceData FS2.

To investigate how interaction with extracellular matrix modulated colony formation, we analyzed the adherence of cells to fibronectin-coated glass surfaces. Surprisingly, cells lacking either STIM isoform but not cells lacking both isoforms had reduced adhesion compared with WT cells (Fig. S1 C). Since adhesion to the extracellular matrix is crucial for cell migration, we used a transwell migration and invasion assays to test how deletion of each STIM isoform affects this cellular activity. Consistent with earlier observations (Yang et al., 2009; Miao et al., 2019), we found a significant decrease in these activities in cells lacking either STIM1 or STIM2 (Fig. 2, A and B; and Fig. S1 D). However, as in the adhesion assay, migration or invasion of two different clones of cells lacking both STIM isoforms was restored (Fig. 2, A and B; and Fig. S1 D). Confirming that changes in cell migration or invasion were linked to the expression of either STIM protein, the re-expression of STIM1 or STIM2 in dKO cells significantly reduced cell migration (Fig. 2, C and D; and Fig. S1 E). This indicates that while STIM proteins are entirely dispensable for cell migration, they play an important regulatory role in this cellular function. We first investigated whether changes in epithelial-to-mesenchymal transition (EMT) or the expression of NFAT1, previously reported to occur following deletion of STIM1 or STIM2 (Miao et al., 2019; Cheng et al., 2022; Hammad et al., 2023), could be the underlying mechanisms behind this observation. However, results from expression analyses of several EMT markers and NFAT1 shown in Fig. S2, A–D ruled out these possibilities. To gain further mechanistic insight into the migration rescue phenomenon, we analyzed actomyosin-based contractility and formation of focal adhesions (FAs). Western blot (WB) analysis using two different antibodies against phosphorylated myosin light chain (pMLC) revealed an increase in the levels of myosin light chain (MLC) phosphorylation in S1KO and S2KO cells compared with WT or dKO cells (Fig. 2, E and F). However, pMLC immunostaining indicated that the relative pMLC levels at cortical regions of the cell, areas marked by F-actin bundles, were reduced in S1KO and S2KO cells compared with WT or dKO cells (Fig. 2, G and H). The arrangement and total content of F-actin remained unchanged (Fig. S2 E). This suggests that the spatial coordination of traction forces is likely disrupted in S1KO and S2KO cells but not in dKO cells. To further assess changes in another migration-related cellular process, we examined the number of focal adhesions (FAs). Consistent with the pMLC staining patterns, paxillin immunostaining showed that deletion of either STIM1 or STIM2 led to a reduction in the average number of FAs, whereas this effect was rescued in dKO cells lacking both STIM isoforms (Fig. 2, I and J). Taken together, the above results indicate that deletion of either STIM1 or STIM2 suppresses mesenchymal cell migration in breast cancer cells, while deletion of both isoforms unexpectedly rescues this deficiency.

### IP3Rs are critical regulators of mesenchymal cell migration in STIM-deficient cells

**Figure S3.**
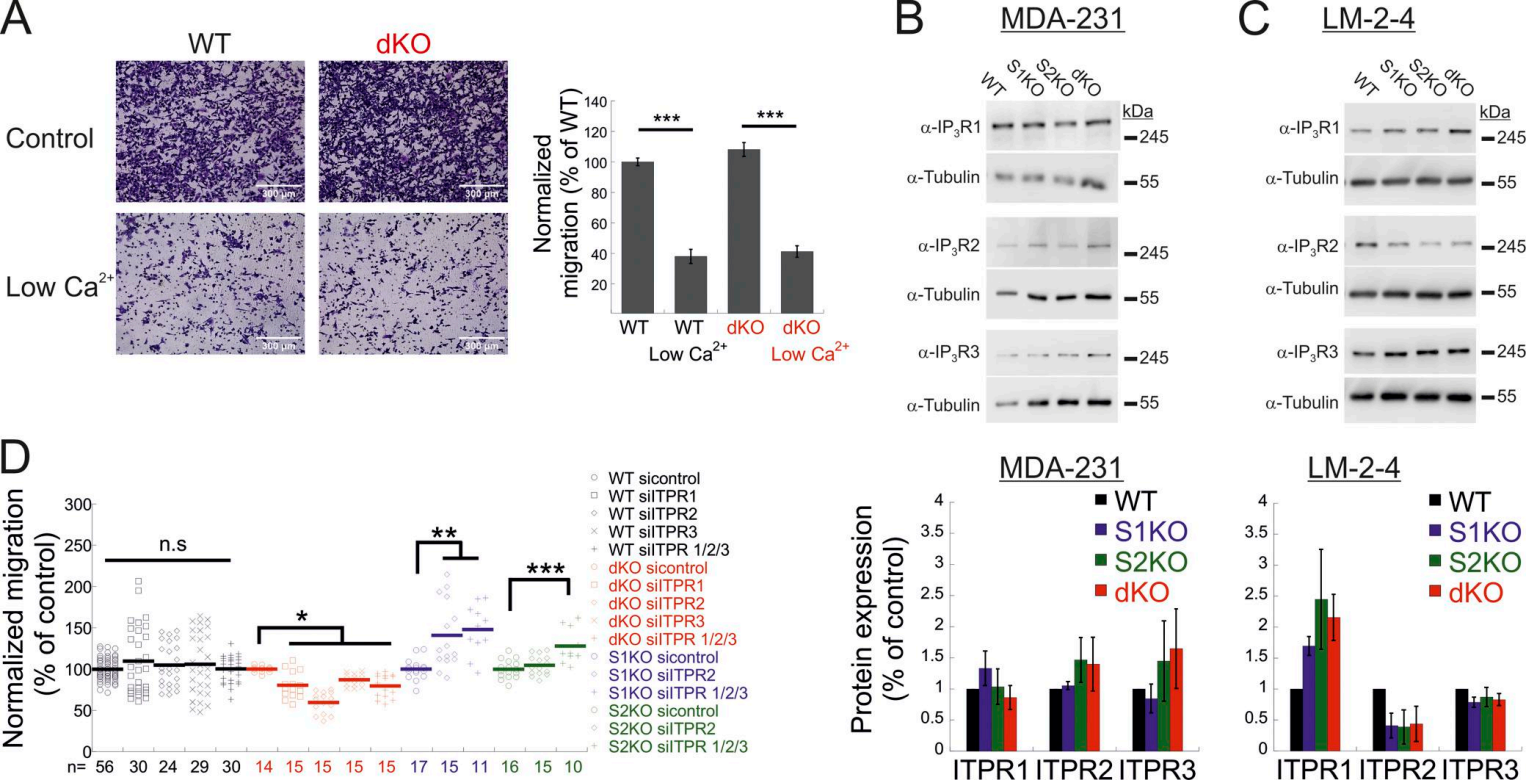
**Additional data related to** Fig. 3 **. (A)** Expression of IP3Rs in MDA-MB-231 and LM2-4 cells. (A-left) Representative images of WT and dKO migrating cells under normal conditions or low Ca^2+^, as indicated. (A-right) Quantification of the number of migrating cells from each group was normalized to that of control (WT) cells. Scale bar = 350 μm. **(B and C)** Representative images (left) and quantitation (right) of WB analysis (*n* = 3–4) of lysates prepared from the indicated MDA-MB-231 (A) or LM2-4 (B) cells using antibodies for IP3R1, IP3R2, IP3R3, or tubulin, as indicated. **(D)** Migration analysis of WT, S1KO, S2KO, or dKO cells treated with the indicated ITPR siRNAs. Bars show the mean ± SEM. Statistics: one-way ANOVA with Tukey’s post hoc test; *P < 0.05, **P < 0.01, ***P < 0.001. Source data are available for this figure: SourceData FS3.

**Figure 3.**
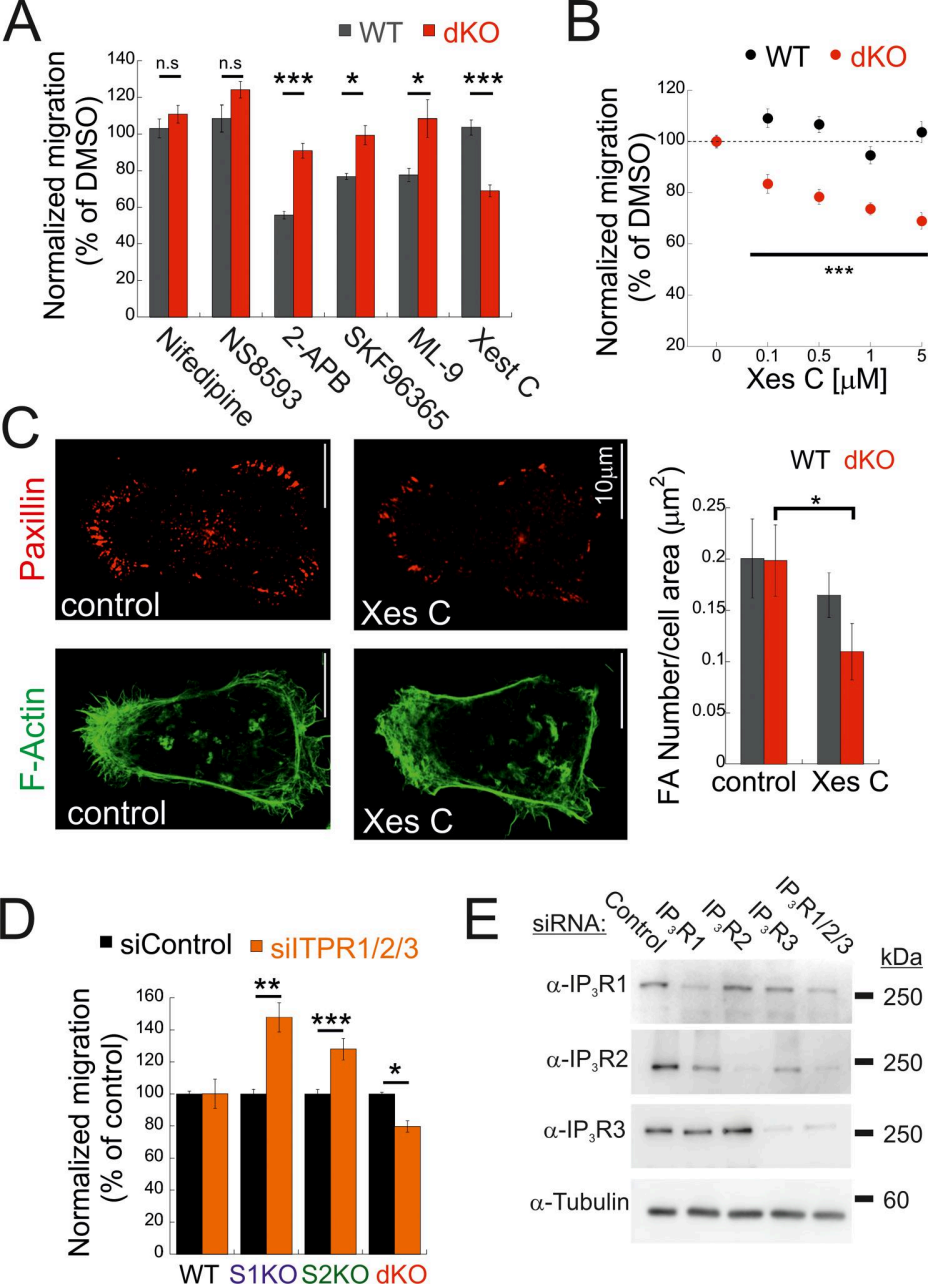
**Deletion of STIM proteins sensitizes cell migration to IP3R inhibition. (A)** Indicated compounds were added to either WT or dKO cells, and the number of migrating cells from each group was normalized to that of control (DMSO-treated cells). The following inhibitor concentrations were used: 2-APB at 50 μM (WT, *n* = 5; dKO, *n* = 11), nifedipine at 50 μM (WT, *n* = 5; dKO, *n* = 10), SKF96365 at 10 μM (WT, *n* = 5; dKO, *n* = 10), Xes-C at 5 μM (WT, *n* = 10; dKO, *n* = 5), NS8593 at 5 μM (WT, *n* = 5; dKO, *n* = 5), and ML-9 at 10 μM (WT, *n* = 5; dKO, *n* = 5). Bars show the mean ± SEM. **(B)** Migration was analyzed as in A using the indicated concentration of Xes-C (WT, *n* = 11–16; dKO, *n* = 5–11). **(C)** (C-left) Representative images (left) of anti-paxillin (red) and phalloidin (F-actin, green) fluorescence in dKO cells treated with Xes-C or DMSO (control). (C-right) Mean ± SEM of FAs per cell in WT (control, *n* = 9; Xes-C, *n* = 15) or dKO (control, *n* = 8; Xes-C, *n* = 13) cells. Scale bar = 10 μm. **(D)** Migration analysis (bars show the mean ± SEM) of WT (*n* = 14), S1KO (*n* = 11), S2KO (*n* = 10), or dKO cells (*n* = 15) treated with the indicated ITPR siRNAs. **(E)** WB analysis using antibodies for IP3R1, IP3R2, IP3R3, or tubulin of lysates prepared from WT cells treated with siRNAs against IP3R1, IP3R2, or IP3R3, as indicated. Statistics: two-tailed *t* test; *P < 0.05, **P < 0.01, ***P < 0.001. Source data are available for this figure: SourceData F3.

We next asked how deletion of both STIM isoforms rescues cell migration. Phosphorylation of MLC and generation of focal adhesions are calcium-regulated processes (Tsai and Meyer, 2012; Tsai et al., 2014; Okeke et al., 2016). Indeed, reduction of extracellular Ca^2+^ levels attenuated migration of both WT and dKO cells (Fig. S3 A). We therefore investigated the involvement of different Ca^2+^ signaling proteins in cell migration. We conducted a pharmacological screen to determine whether a Ca^2+^ permeation or release pathway contributed to the restoration of cell migration in STIM dKO cells. In both WT and dKO cells, migration was insensitive to treatment with either nifedipine, a blocker of voltage-dependent calcium channels, or NS8593, a blocker of K_Ca2+_ and TRPM7 channels. Consistent with the involvement of SOCE in cell migration of WT cells but not in STIM dKO cells, application of either SKF96365, 2-APB, or ML-9, compounds with a shared inhibitory effect on SOCE, inhibited cell migration in WT but not dKO cells (Fig. 3 A). Notably, treatment with xestospongin C (Xes-C), a selective inhibitor of IP3Rs, inhibited the migration of STIM dKO cells but not WT cells. Examination of the dose-dependent effects of Xes-C on inhibiting cell migration indicated that deletion of both STIM isoforms led to increased sensitivity to IP3R inhibition as compared to WT cells (Fig. 3 B). Consistent with the effect of IP3R inhibition on cell migration in dKO cells, treatment with Xes-C reduced the number of FAs in dKO cells but not in WT cells (Fig. 3 C). To validate that the effect of Xes-C treatment on cell migration was mediated by IP3Rs, we treated either WT, S1KO, S2KO, or dKO cells with siRNAs against the three IP3R isoforms. WB analysis shown in Fig. 3 E indicates that treatment with siRNAs against IP3R1-3 silenced the expression of each IP3R by >70% (73.5 ± 4% for IP3R1, 75.7 ± 16% for IP3R2, and 83.9 ± 1% for IP3R3). Remarkably, analysis of cell migration in siRNA-treated cells revealed that while WT cells were statistically unaffected, migration was increased in S1KO and S2KO cells but decreased in STIM dKO cells (Fig. 3 D and Fig. S3 D). Analysis of IP3R expression in WT, S1KO, S2KO, and dKO cells showed that the degree of expression in either cell type was not significantly changed (Fig. S3 B). Taken together, the above results reveal that IP3Rs mediate bidirectional effects on cell migration depending on the pattern of STIM protein expression.

### STIM proteins facilitate a diffuse mode of Ca^2+^ release via IP3Rs

**Figure 4.**
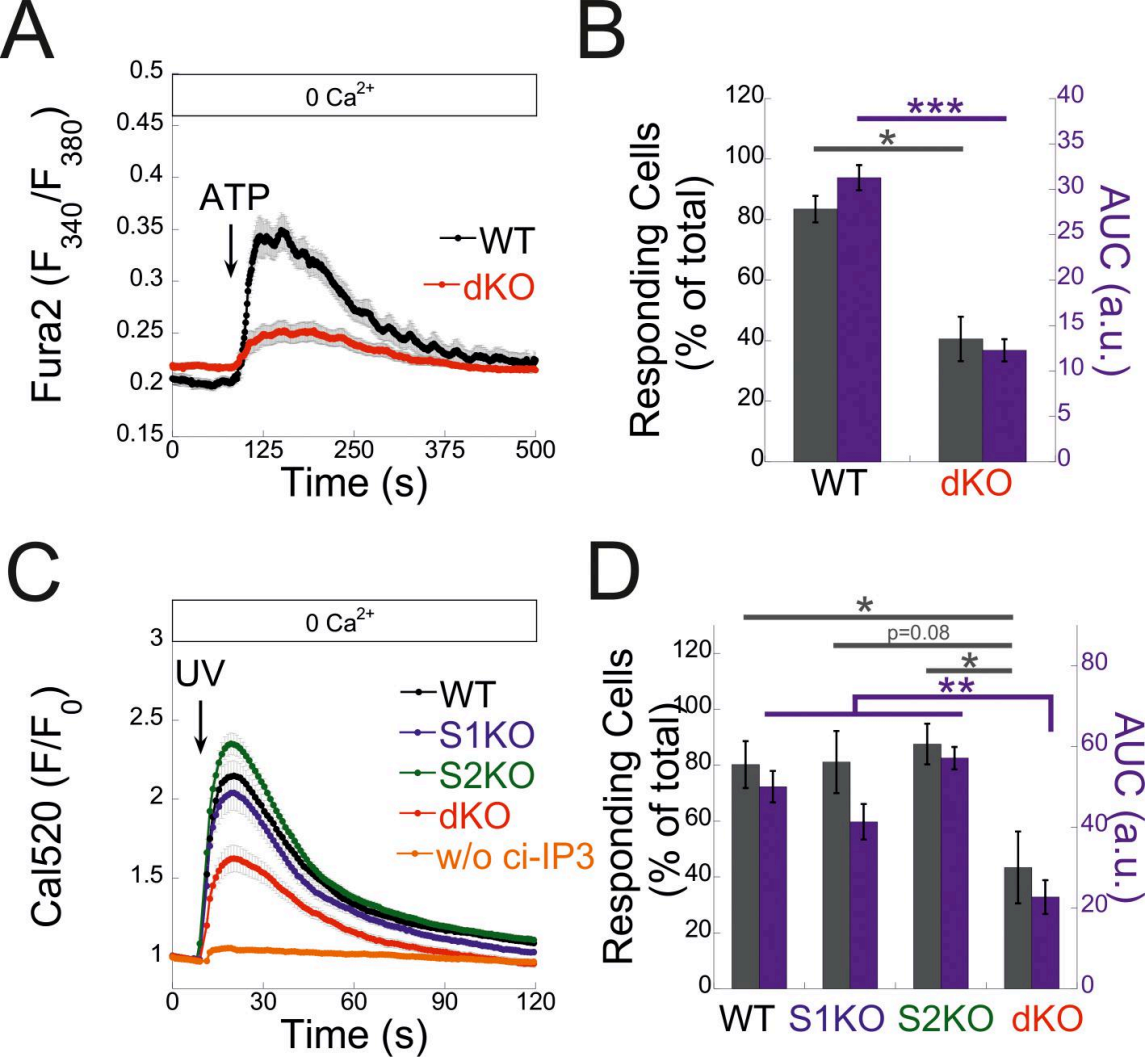
**Deletion of both STIM proteins diminishes the overall IP3-mediated response. (A)** Average intracellular Ca^2+^ responses following application of Ca^2+^-free solution containing 200 μM ATP in a representative experiment are shown for WT (*n* = 48) and dKO (*n* = 46) cells. **(B)** Bars show the mean ± SEM of the percentage of responding cells (gray bars) or quantitation of ATP-induced Ca^2+^ release (AUC, purple bars) from three experiments as shown in A. **(C)** Cells were loaded with ci-IP3 and Cal-590 (see Materials and methods). Panel shows the average intracellular Ca^2+^ responses for WT (*n* = 163), S1KO (*n* = 139), S2KO (*n* = 200), or dKO (*n* = 189) cells following a brief (1-s) illumination with 405-nm laser. Note the lack of response in WT cells lacking ci-IP3 (*n* = 19). **(D)** Bars show the mean ± SEM of the percentage of responding cells (gray bars) or quantitation of UV-induced Ca^2+^ release (AUC, purple bars) from seven experiments as shown in C. Statistics: two-tailed *t* test (B) and one-way ANOVA with Tukey’s post hoc test (D); *P < 0.05, **P < 0.01, ***P < 0.001. AUC, area under the curve.

**Figure S4.**
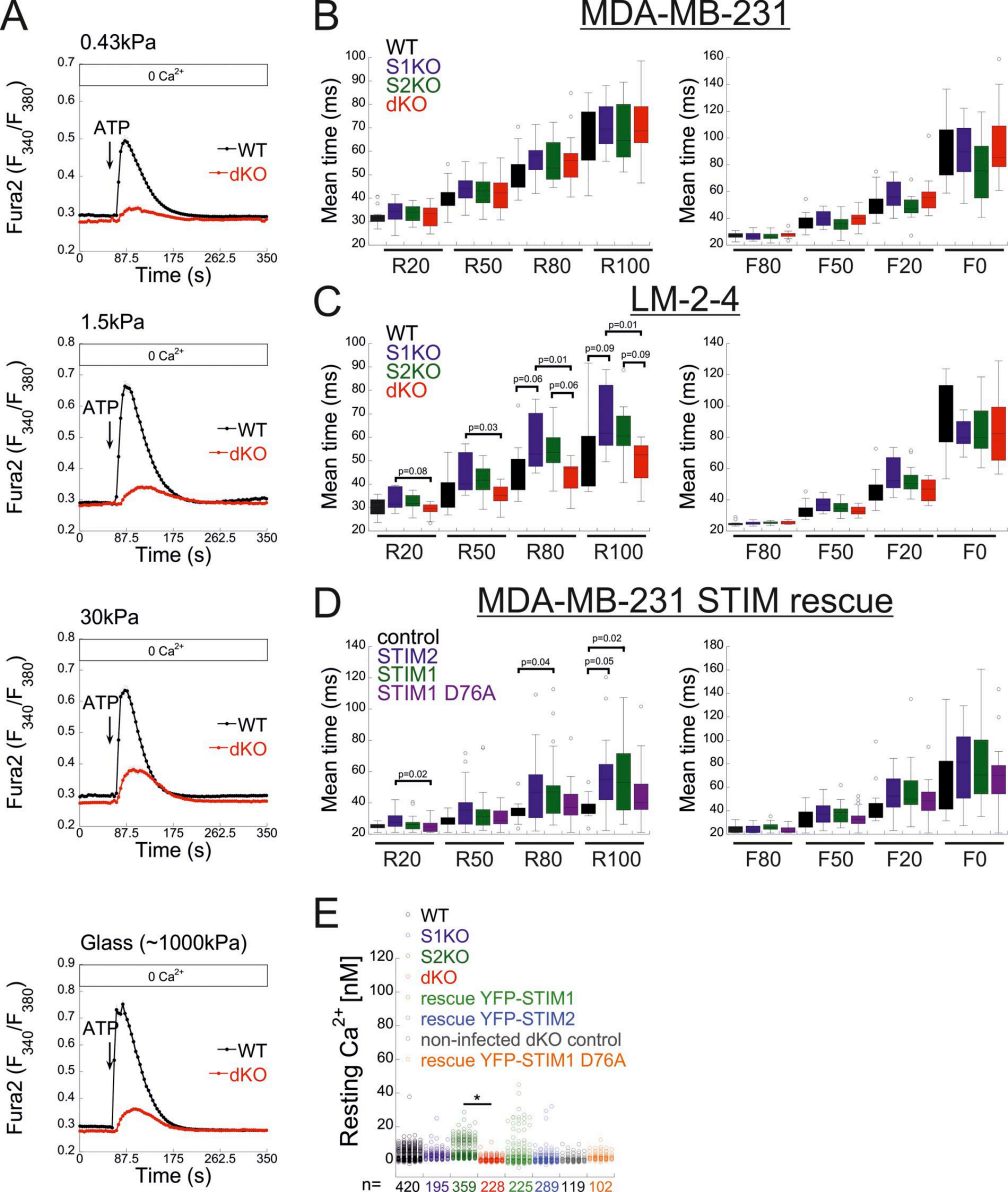
**Additional data related to** Figs. 4, 5 and 6**. (A)** Cells were cultured on Matrigel-coated polyacrylamide gels of the indicated stiffness or on glass. The average intracellular Ca^2+^ responses in Fura-2–loaded cells following the application of a Ca^2+^-free solution containing 200 µM ATP are shown for each condition (*n* = 202 for both WT and dKO cells). **(B–D)** Mean rise and decay times of Ca^2+^ puff fluorescence were measured during increases and decreases to 20%, 50%, 80%, and 100%, analyzed from the indicated type of MDA-MB-231 (B) or LM2-4 (C) cells shown in Fig. 5 or from MDA-MB-231 STIM dKO control or STIM1, STIM2, or STIM1 D76A–expressing (rescue) cells (D) shown in Fig. 6. **(E)** Quantification of resting Ca^2+^ levels in the indicated cells following loading with a Ca^2+^ indicator (Fura-2), caged IP3 (ci-IP3/PM), and EGTA-AM. Statistics: one-way ANOVA with Tukey’s post hoc test; *P < 0.05.

**Figure 5.**
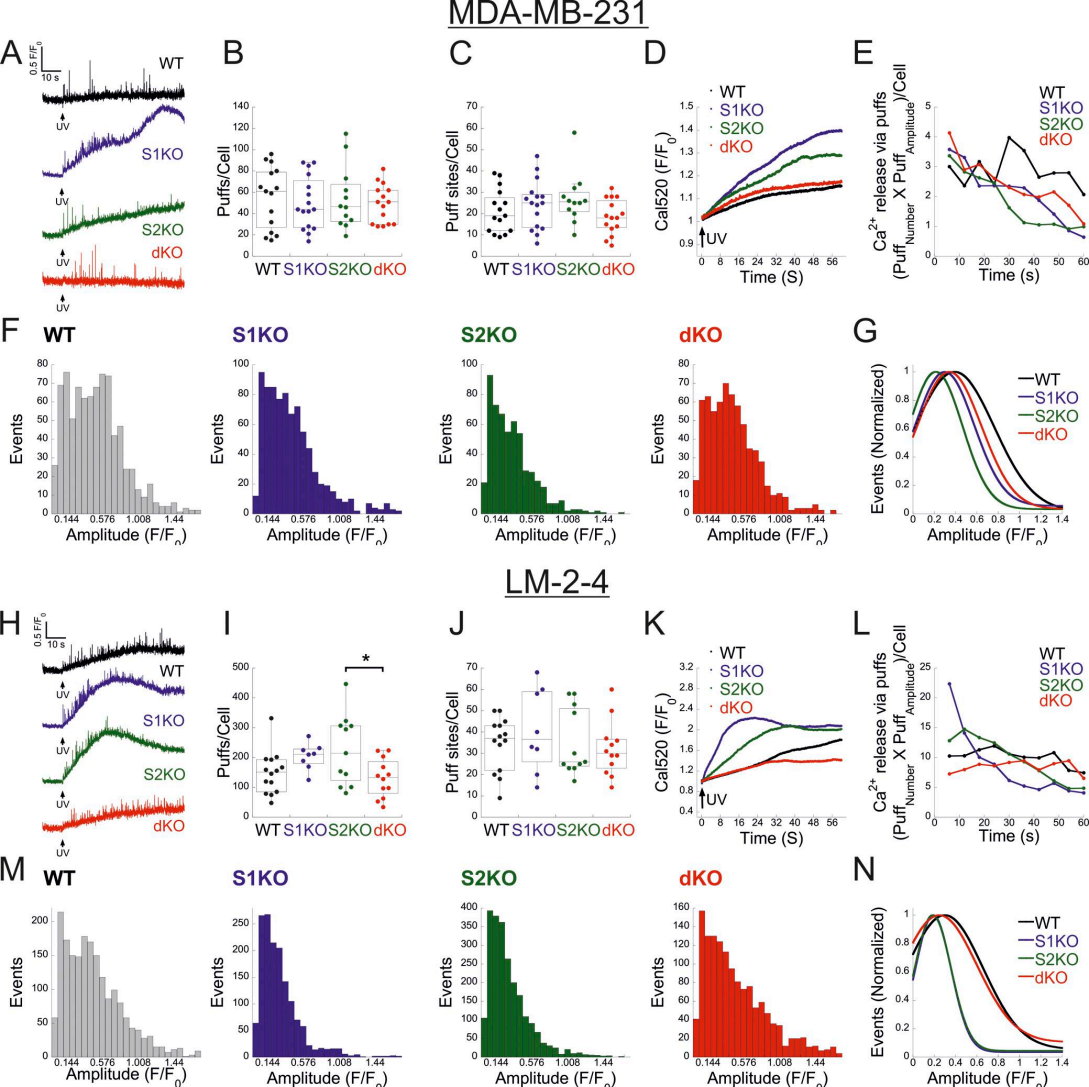
**STIM proteins promote a change in the IP3R mode of Ca** ^**2+**^ **release.** Analysis of global and puff Ca^2+^ release in the indicated MDA-MB-231 (WT, *n* = 15; S1KO, *n* = 16; S2KO, *n* = 12; dKO, *n* = 15) cells or LM2-4 (WT, *n* = 14; S1KO, *n* = 8; S2KO, *n* = 11; dKO, *n* = 12) cells. **(A and H)** Representative traces of Cal-520 fluorescence ratios (ΔF/F0) recorded from the center of an individual puff site before and after ci-IP3 uncaging by a pulse (1 s) of 405-nm light in the indicated MDA-MB-231 (A) or LM2-4 (H) cells. **(B, C, I, and J)** Boxplot analysis shows the number of Ca^2+^ puffs (B and I) or puff sites (C and J) per cell for the indicated type of MDA-MB-231 (B and C) or LM2-4 (I and J) cells. **(D and K)** Average global Ca^2+^ responses recorded after UV-induced IP3 uncaging for the indicated type of MDA-MB-231 (D) or LM2-4 (K) cells. **(E and L)** Time course of Ca^2+^ release via Ca^2+^ puff in the indicated MDA-MB-231 (E) or LM2-4 (L) cells was quantified by multiplying the number of Ca^2+^ puffs by the average puff amplitude within 6-s time intervals. Note that in S1KO or S2KO cells, the rise in cell-wide Ca^2+^ (D and K) occurs, while localized Ca^2+^ release decreases (E and L). **(F, G, M, and N)** Amplitude distributions of local Ca^2+^ puffs in the indicated MDA-MB-231 (F) or LM2-4 (M). **(G and N)** Curves show the normalized Gaussian fit to the data shown in F or M for the indicated type of cells. Statistics: one-way ANOVA with Tukey’s post hoc test; *P < 0.05.

**Figure S5.**
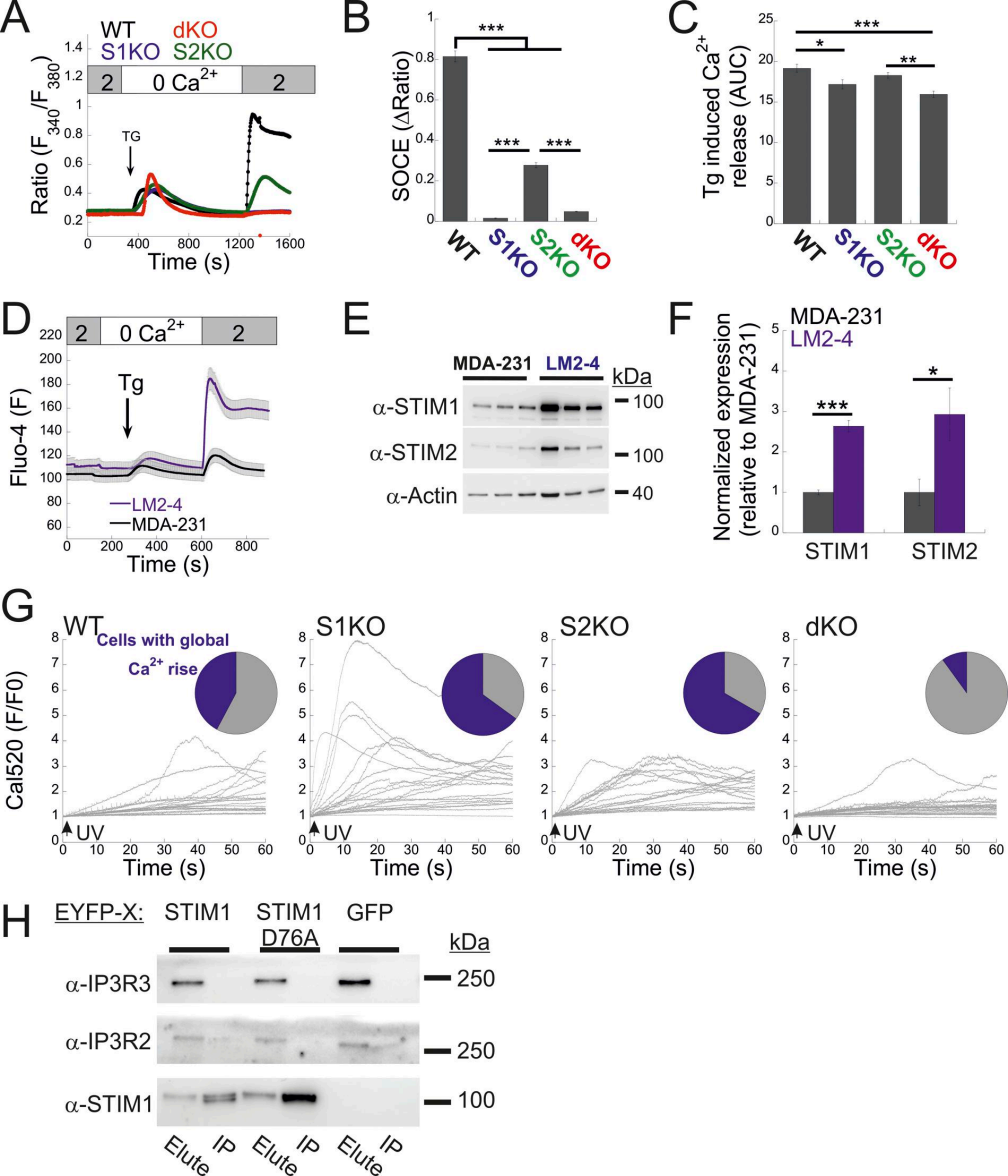
**Analysis of STIM expression and function in LM2-4 cells. (A)** Average intracellular Ca^2+^ responses during SOCE are shown for WT (*n* = 137), S1KO (*n* = 70), S2KO (*n* = 73), and S1/S2 dKO (*n* = 87) LM2-4 cells. **(B and C)** Quantification of SOCE (B) and Tg-induced ER Ca^2+^ release (C) for individual cells depicted in A. The AUC was calculated from 400 to 1,200 s. **(D)** Analysis of SOCE in Fluo-4–loaded LM2-4 cells (*n* = 46) compared with MDA-MB-231 cells (*n* = 47). **(E and F)** Representative images (left) and quantification (right) of WB analysis (*n* = 3–4) of lysates from LM2-4 and MDA-MB-231 cells using antibodies against STIM1, STIM2, or actin, as indicated. **(G)** Traces of cell-wide Ca^2+^ dynamics recorded from individual WT, S1KO, S2KO, and dKO LM2-4 cells using the experimental protocol described in Fig. 5. Upper inset shows a pie chart of the fraction of cells showing global Ca^2+^ rise (blue) following UV-induced uncaging of IP3. **(H)** Total cell lysates were prepared from S1/S2 dKO HEK293 cells expressing either EYFP-STIM1 WT, the D76A mutant, or EGFP. WB images of the IP protein material and eluted fractions are shown, demonstrating the absence of interaction between STIM1 and IP3R2 or IP3R3. Bars show the mean ± SEM. Statistics: one-way ANOVA with Tukey’s post hoc test (B and C) or two-tailed *t* test (F); *P < 0.05, **P < 0.01, ***P < 0.001. AUC, area under the curve; IP, immunoprecipitated. Source data are available for this figure: SourceData FS5.

**Figure 6.**
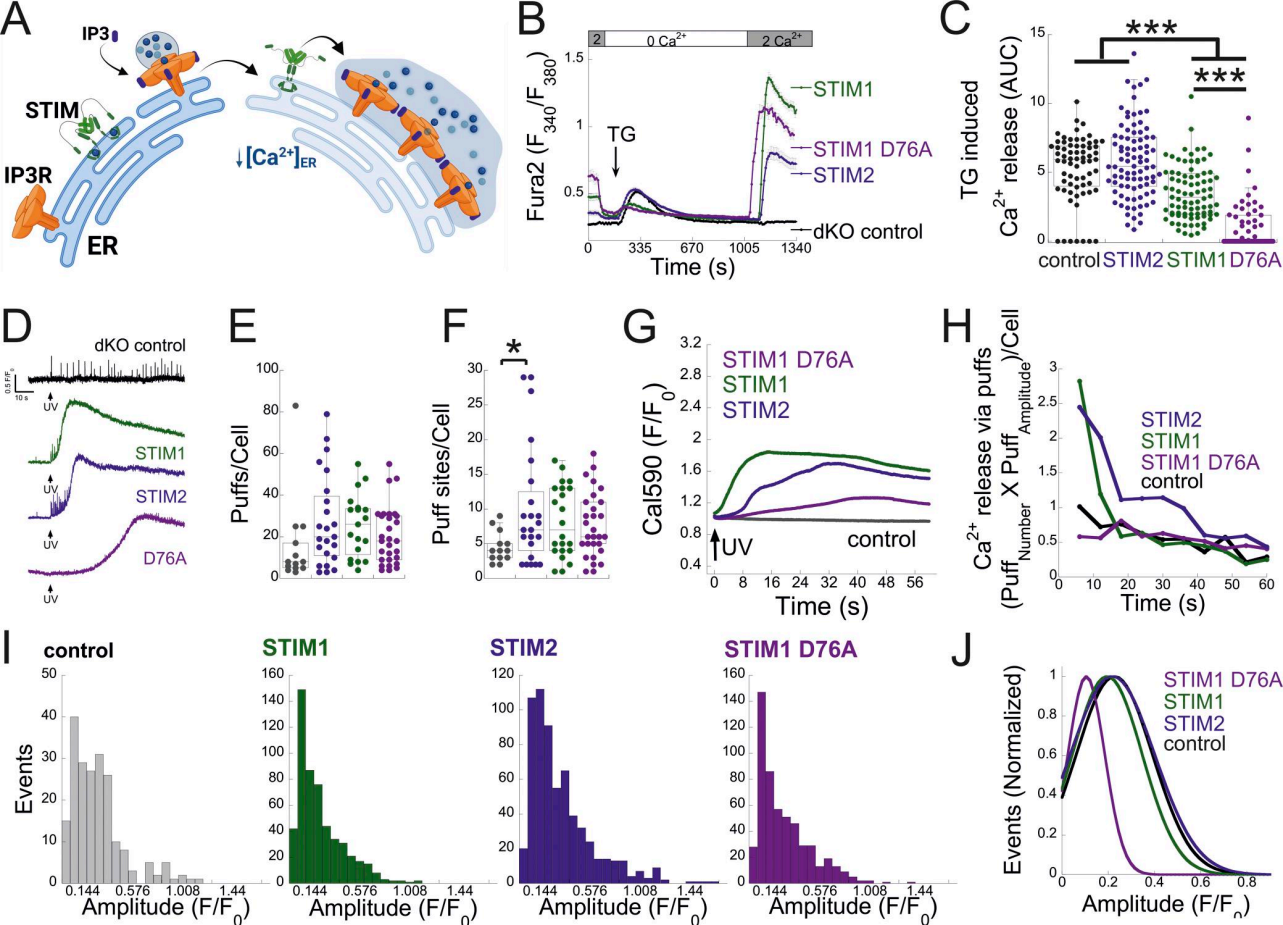
**Restoring STIM1 or STIM2 expression rescues IP3-dependent Ca** ^**2+**^ **release through a diffuse release mode. (A)** Proposed model for STIM regulation of diffuse Ca^2+^ release via IP3R. Under ER Ca^2+^ replete conditions, STIM is at a resting conformation and IP3R-mediated Ca^2+^ release occurs primarily via local Ca^2+^ puffs. Upon a decrease in [Ca^2+^]_ER_, STIM adopts an active conformation and promotes a diffuse mode of Ca^2+^ release via IP3R. **(B)** Average (mean ± SEM) intracellular Ca^2+^ responses of basal and SOCE are shown for control dKO (*n* = 67) or for dKO cell expressing STIM1 (*n* = 85), STIM2 (*n* = 89), or STIM1 D76A (*n* = 58) cells. **(C)** Boxplot shows quantification of Tg-induced ER Ca^2+^ release for individual cells shown in B. **(D–J)** Analysis of global and puff Ca^2+^ release in MDA-MB-231 control dKO cells (*n* = 13) or in cells expressing EYFP-STIM1 (*n* = 19), EYFP-STIM2 (*n* = 23), or EYFP-STIM1 D76A (*n* = 29). **(D)** Representative traces of Cal-590 fluorescence ratios (ΔF/F0) recorded from the center of an individual puff site before and after ci-IP3 uncaging by a pulse (1 s) of 405-nm light in the indicated cells. **(E and F)** Boxplot shows the number of Ca^2+^ puffs (E) or puff sites (F) per cell for the indicated cells. **(G)** Average global Ca^2+^ responses recorded after UV-induced IP3 uncaging for the indicated cells. **(H)** Time course of Ca^2+^ release via Ca^2+^ puff in the indicated cells was quantified as in Fig. 5 E. Note that in cells expressing STIM1 or STIM2, a rise in global Ca^2+^ levels (G) is accompanied by a decrease in localized Ca^2+^ release (H). However, in cells expressing the STIM1 D76A mutant, the global Ca^2+^ increase occurs without a corresponding decrease in localized release. **(I)** Amplitude distributions of local Ca^2+^ puffs in the indicated cells. **(J)** Curves show the normalized Gaussian fit to the data shown in I for the indicated type of cells. Statistics: one-way ANOVA with Tukey’s post hoc test; *P < 0.05, ***P < 0.001.

We next investigated the functional crosstalk between STIMs and IP3Rs. Stimulation of Fura-2–loaded cells with ATP indicated diminished Ca^2+^ release in STIM dKO as compared to WT cells (Fig. 4 A). This reduction was observed under both soft and stiff extracellular environments (Fig. S4 A). While the lower ER Ca^2+^ content in STIM dKO cells (Fig. 1, C and D) likely contributed to this decrease, analysis of the fraction of responding cells showed a significant reduction in STIM dKO cells compared with WT cells (Fig. 4 B). A similar trend was observed in HEK293 cells lacking both STIM isoforms when stimulated with carbachol, despite no changes in resting ER calcium levels (Emrich et al., 2021). This led us to consider the possibility that the observed changes in MDA-MB-231 cells might arise from alterations in the IP3 signal transduction pathway upstream of the IP3R. To bypass the receptor–phospholipase C machinery, we co-loaded WT, S1KO, S2KO, or STIM dKO cells with Ca^2+^ indicator (Cal-520) and caged IP3 (ci-IP3/PM) and used photo-uncaging of ci-IP3 to directly stimulate Ca^2+^ release via IP3Rs. As expected, a brief (1-s) pulse of 405-nm light induced significant Ca^2+^ release in cells loaded with ci-IP3 but not in control cells (Fig. 4 C). The average IP3R-mediated Ca^2+^ release was lower in dKO cells as compared to all other groups, suggesting that deletion of both STIM1 and STIM2 attenuated Ca^2+^ release via IP3Rs (Fig. 4 C). Consistent with ATP-induced Ca^2+^ release, analysis of the fraction of responding cells indicated a reduction in STIM dKO cells as compared to all other groups (Fig. 4 D). Since the IP3-induced global Ca^2+^ signals originate from localized Ca^2+^ puffs that recruit additional IP3Rs via Ca^2+^-induced Ca^2+^ release, we considered the possibility that deletion of STIM proteins affected Ca^2+^ puff via IP3Rs. To address this possibility, cells were co-loaded with ci-IP3 and EGTA, to maintain resting Ca^2+^ under 50 nM in all cell types (Fig. S4 E) and attenuate the spread of Ca^2+^ between neighboring IP3R clusters, and Ca^2+^ puffs were recorded using total internal reflection microscopy at a rate of 50 frames/s for 60 s following ci-IP3 uncaging (Fig. 5 A; and Videos 1, 2, 3, and 4). Analysis of Ca^2+^ puffs showed that deletion of either STIM protein alone or together had no significant effect on the mean number of puff events, on release sites, nor on the puff mean rise or fall times (Fig. 5, A–C and Fig. S4 B). Remarkably, the average global Ca^2+^ signal under these conditions was higher in S1KO or S2KO cells as compared to WT or dKO cells (Fig. 5 D). Notably, estimation of global and puff-induced Ca^2+^ release showed that while the global Ca^2+^ released was higher in S1KO (225.8 ± 25%) or S2KO (179.9 ± 23%) cells as compared to WT (100 ± 12.8%) or dKO (125.6 ± 20%) cells, Ca^2+^ release via puff showed a gradual decrease over time in STIM-deficient cells (Fig. 5 E). Moreover, analysis of puff amplitude distribution showed that deletion of either STIM1 or STIM2 reduced the median puff amplitude as compared to WT or STIM dKO cells (Fig. 5, F and G). Consistent with previous findings (Lock and Parker, 2020), this suggests that puff activity in S1KO or S2KO cells contributes only a small fraction of the total global Ca^2+^ signal, which arises primarily from an increase in the diffuse mode of Ca^2+^ release by IP3Rs. We next asked whether the above findings represent a special case for MDA-MB-231 cells. Deletion of STIM1 and STIM2, either individually or together, in the isogenic lung derivative and highly metastatic breast cancer cell line LM2-4 revealed that the observed phenomena were not unique to MDA-MB-231 cells. As demonstrated in Fig. S1 B and Fig. S5, A–C, deletion of STIM2 in LM2-4 cells resulted in a mild reduction in SOCE, whereas deletion of STIM1 alone or together with STIM2 abolished SOCE (Fig. S5, A and B). A comparable pattern was observed for the average levels of Ca^2+^ released from the ER by Tg (Fig. S5 C), indicating higher resting ER luminal Ca^2+^ levels in WT cells compared with STIM1-deficient cells (Fig. S5 C). Although the analysis of IP3-evoked Ca^2+^ signals showed a greater number of puff sites and events per cell in LM2-4 cells compared with MDA-MB-231 cells, the deletion of STIM isoforms resulted in similar trends in both cell lines (Fig. 5, A–C and H–J; and Videos 1, 2, 3, 4, 5, 6, 7, and 8). While a slight increase in puff events was observed in S2KO cells, the deletion of STIM1 or both STIM1 and STIM2 did not significantly alter the number of Ca^2+^ puff sites or events. Importantly, consistent with results in MDA-MB-231 cells, the deletion of STIM1 or STIM2, but not both, in LM2-4 cells reduced the median puff amplitude and enhanced diffuse Ca^2+^ release via IP3Rs (Fig. 5, K–N). WB analysis of the three IP3R isoforms in LM2-4 cells indicated that while IP3R3 expression remained unchanged following STIM deletion, IP3R1 expression increased and IP3R2 expression decreased (Fig. S3 C). These findings suggest that similar to MDA-MB-231 cells, the changes in IP3R function observed in STIM-deficient LM2-4 cells are not attributable to alterations in IP3R expression levels. Taken together, results from both MDA-MB-231 and LM2-4 cells indicate that diffuse Ca^2+^ release via IP3Rs is stimulated by either STIM isoform. An apparent puzzling observation in both cell types, however, is that this regulatory function is largely absent in WT cells that express both STIM isoforms. Since deletion of either STIM isoform has been shown to induce transcriptional remodeling in various cell types, including MDA-MB-231 cells (Hammad et al., 2023), we hypothesized that different changes in the expression of one or more IP3R regulatory components might account for the observed alterations in IP3R function. Alternatively, since STIM function is regulated by luminal ER Ca^2+^, which is reduced in the STIM-deficient cells compared with WT cells (Fig. 1, C and D; and Fig. S5 C), we also considered the possibility, as illustrated in Fig. 6 A, that the decrease in ER Ca^2+^ levels in S1KO or S2KO cells partially activated the remaining STIM isoform, thereby influencing IP3R activity. To examine these possibilities, we tested whether reintroduction of STIM1 or STIM2 expression in MDA-MB-231 STIM dKO cells would restore the IP3-dependent diffuse Ca^2+^ signal. As anticipated, analysis of Ca^2+^ entry in STIM dKO cells ectopically expressing EYFP-tagged STIM1, STIM2, or the constitutively active STIM1 D76A mutant demonstrated robust Ca^2+^ entry after ER Ca^2+^ depletion (Fig. 6 B). Indicative of desensitization to changes in ER Ca^2+^ levels of the STIM1 D76A mutant, robust Ca^2+^ entry was observed in cells expressing the mutant even before ER Ca^2+^ depletion (Fig. 6 B). Estimation of resting ER Ca^2+^ levels showed that while the re-expression of STIM2 had no effect, the expression of WT STIM1 led to about ∼40% reduction, while the expression of STIM1 D76A led to about ∼70% reduction in ER Ca^2+^ levels as compared to control STIM dKO cells (Fig. 6 C). Analysis of Ca^2+^ puff indicated that the average number of puff sites or events per cell was increased in the STIM-expressing cells; however, for the most part this increase was not statistically significant (Fig. 6, D–F). Importantly, the global Ca^2+^ signal was higher in either one of the STIM-expressing groups as compared to control cells (Fig. 6 G; and Videos 9, 10, and 11). The difference was less pronounced in cells expressing D76A STIM1 than STIM1-expressing cells, likely owing to the lower ER Ca^2+^ levels in the mutant-expressing cells (Fig. 6 C). Notably, analysis of the total Ca^2+^ released over time via Ca^2+^ puffs showed that the expression of STIM1 or STIM2 but not STIM1 D76A leads to a gradual decrease in puff-induced Ca^2+^ release (Fig. 6 H). A decrease in the median puff amplitude was observed in cells expressing STIM1 D76A and to a lesser extent in cells expressing STIM1 compared with control (STIM dKO) or STIM2-expressing cells (Fig. 6, I and J). Taken together, these findings therefore argue against the STIM-induced transcriptional remodeling hypothesis and indicate that the expression of either STIM isoform is sufficient to promote a diffuse Ca^2+^ release via IP3Rs. Furthermore, since the D76A mutation stabilizes STIM1 in an active conformation, the findings also suggest that facilitation of an IP3R diffuse Ca^2+^ release by STIM proteins occurs when STIMs transition from a resting to active conformation.

**Video 1.** **MDA-MB-231 (WT).** Time-lapse TIRFM of WT MDA-MB-231 cells co-loaded with Cal-520 AM, EGTA-AM, and caged-IP3. IP3 was uncaged at 10 s (frame 500) using a 405-nm laser pulse. Images were acquired at 50 frames per second. This video relates to Fig. 5.

**Video 2.** **MDA-MB-231 STIM1 KO (S1KO).** Time-lapse TIRFM of S1KO MDA-MB-231 cells co-loaded with Cal-520 AM, EGTA-AM, and caged-IP3. IP3 was uncaged at 10 s (frame 500) using a 405-nm laser pulse. Images were acquired at 50 frames per second. This video relates to Fig. 5.

**Video 3.** **MDA-MB-231 STIM2 KO (S2KO).** Time-lapse TIRFM of S2KO MDA-MB-231 cells co-loaded with Cal-520 AM, EGTA-AM, and caged-IP3. IP3 was uncaged at 10 s (frame 500) using a 405-nm laser pulse. Images were acquired at 50 frames per second. This video relates to Fig. 5.

**Video 4.** **MDA-MB-231 STIM1/STIM2 dKO.** Time-lapse TIRFM of dKO MDA-MB-231 cells co-loaded with Cal-520 AM, EGTA-AM, and caged-IP3. IP3 was uncaged at 10 s (frame 500) using a 405-nm laser pulse. Images were acquired at 50 frames per second. This video relates to Fig. 5.

**Video 5.** **LM2-4 (WT).** Time-lapse TIRFM of WT LM2-4 cells co-loaded with Cal-520 AM, EGTA-AM, and caged-IP3. IP3 was uncaged at 10 s (frame 500) using a 405-nm laser pulse. Images were acquired at 50 frames per second. This video relates to Fig. 5.

**Video 6.** **LM2-4 STIM1 KO (S1KO).** Time-lapse TIRFM of S1KO LM2-4 cells co-loaded with Cal-520 AM, EGTA-AM, and caged-IP3. IP3 was uncaged at 10 s (frame 500) using a 405-nm laser pulse. Images were acquired at 50 frames per second. This video relates to Fig. 5.

**Video 7.** **LM2-4 STIM2 KO (S2KO).** Time-lapse TIRFM of S2KO LM2-4 cells co-loaded with Cal-520 AM, EGTA-AM, and caged-IP3. IP3 was uncaged at 10 s (frame 500) using a 405-nm laser pulse. Images were acquired at 50 frames per second. This video relates to Fig. 5.

**Video 8.** **LM2-4 STIM1/STIM2 dKO.** Time-lapse TIRFM of dKO LM2-4 cells co-loaded with Cal-520 AM, EGTA-AM, and caged-IP3. IP3 was uncaged at 10 s (frame 500) using a 405-nm laser pulse. Images were acquired at 50 frames per second. This video relates to Fig. 5.

**Video 9.** **MDA-MB-231 dKO + EYFP-STIM1.** Time-lapse TIRFM of dKO MDA-MB-231 cells expressing EYFP-STIM1 and co-loaded with Cal-520 AM, EGTA-AM, and caged-IP3. IP3 was uncaged at 10 s (frame 500) using a 405-nm laser pulse. Images were acquired at 50 frames per second. This video relates to Fig. 6.

**Video 10.** **MDA-MB-231 dKO + EYFP-STIM2.** Time-lapse TIRFM of dKO MDA-MB-231 cells expressing EYFP-STIM2 and co-loaded with Cal-520 AM, EGTA-AM, and caged-IP3. IP3 was uncaged at 10 s (frame 500) using a 405-nm laser pulse. Images were acquired at 50 frames per second. This video relates to Fig. 6.

**Video 11.** **MDA-MB-231 dKO + EYFP-STIM1 D76A.** Time-lapse TIRFM of dKO MDA-MB-231 cells expressing EYFP-STIM1 D76A and co-loaded with Cal-520 AM, EGTA-AM, and caged-IP3. IP3 was uncaged at 10 s (frame 500) using a 405-nm laser pulse. Images were acquired at 50 frames per second. This video relates to Fig. 6.

## Discussion

**Figure 7.**
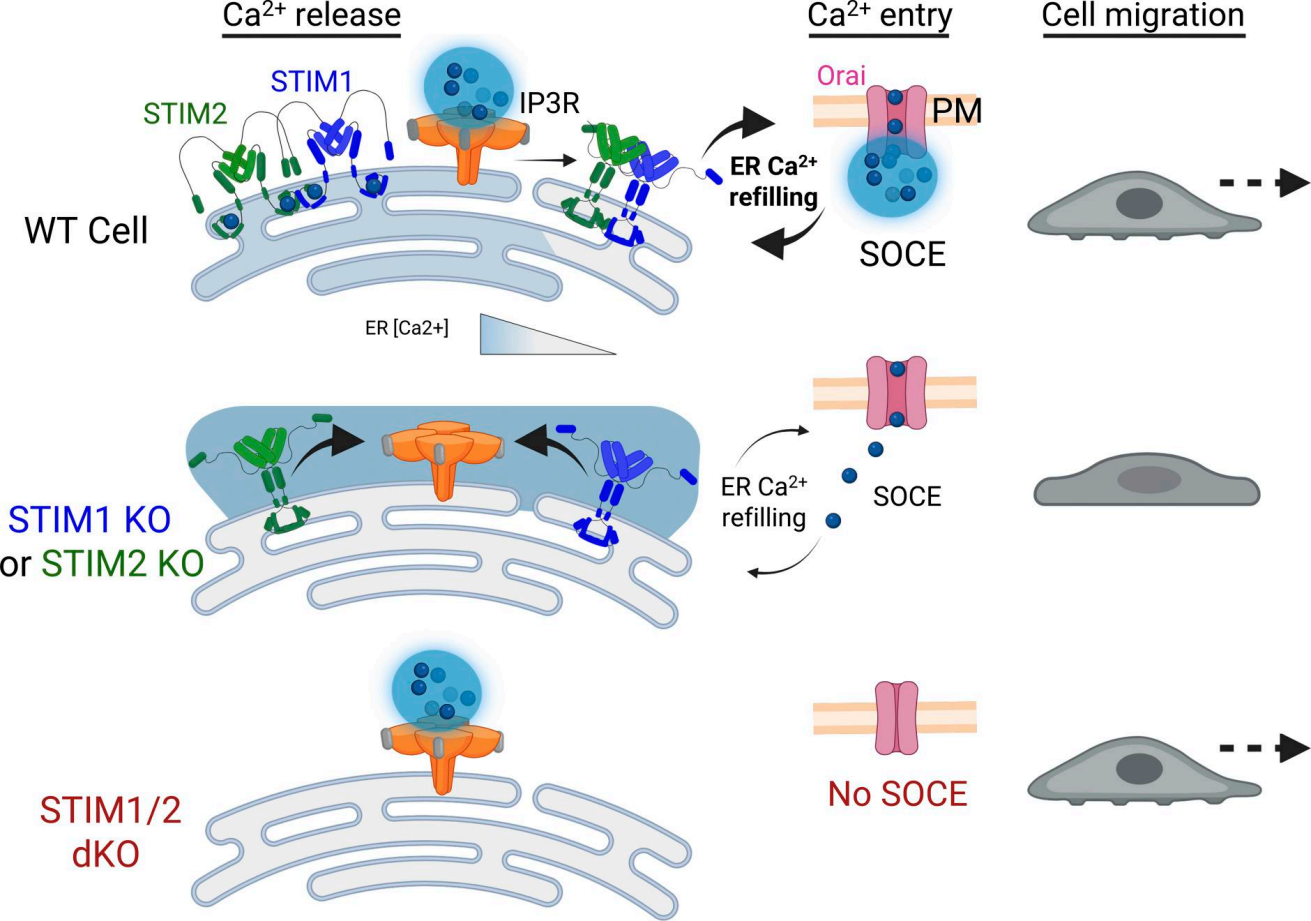
**Mechanistic model of STIM-IP3R crosstalk in regulating breast cancer cell migration.** Breast cancer cell migration is regulated by a dynamic interplay between STIM proteins and IP3Rs, which together shape the spatiotemporal pattern of intracellular Ca^2+^ signaling. In WT cells, transient ER Ca^2+^ depletion triggered by localized IP3R-mediated Ca^2+^ release is rapidly replenished through SOCE, driven by the coordinated activity of both STIM1 and STIM2. This balance of spatiotemporally separated localized Ca^2+^ signals from both IP3Rs and SOCE supports efficient cell migration. In cells lacking either STIM1 or STIM2, the remaining isoform functions near its activation threshold due to lower ER Ca^2+^ stores. As a result, even slight ER Ca^2+^ depletion, such as that initiated by IP3R activity, activates the residual STIM protein, shifting IP3R-mediated Ca^2+^ signals from localized to diffuse/global Ca^2+^ release mode. This altered Ca^2+^ pattern disrupts the finely tuned signaling required for cell migration, leading to impaired motility. In cells lacking both STIM isoforms, this feedback mechanism is lost entirely, allowing IP3Rs to continue generating localized Ca^2+^ signals unmodulated by STIM proteins. Restoration of spatially confined Ca^2+^ signaling supports the maintenance of cell migration despite the complete loss of STIM-mediated SOCE. Created with https://BioRender.com.

In this study, we employed breast cancer cells lacking either or both STIM isoforms to investigate the role of STIM proteins in breast cancer tumorigenesis. Findings from cells lacking both STIM isoforms indicate that while STIM proteins are not obligatory for cell proliferation or migration, they are essential for cell seeding on soft tissue. However, we note that since this function of STIM does not extend to colorectal cancer cells (Pathak et al., 2023, *Preprint*), it is likely a cell type–specific phenomenon. Considering the extensively documented role of SOCE in cell migration, particularly in breast cancer cells (Yang et al., 2009; Mound et al., 2017; Miao et al., 2019; Cheng et al., 2022), it was anticipated that deletion of either of the two STIM isoforms would lead to inhibition of cell migration, which was indeed observed in our findings. However, it was surprising to find that the migration of cancer cells lacking both STIM isoforms was hardly affected. Our efforts to resolve this puzzling observation uncovered a crosstalk between STIM proteins and IP3Rs that is crucial for regulating migration of breast cancer cells, as illustrated in the model shown in Fig. 7. Results from analyses of the effects of IP3R inhibition on cell migration combined with studies of the spatial pattern of IP3-induced Ca^2+^ signals suggest that localized and global Ca^2+^ responses via IP3Rs have opposing influences on cell migration. These findings align with extensive research demonstrating that the specific spatiotemporal organization of calcium signals is essential for coordinating cell migration (Tsai and Meyer, 2012; Wei et al., 2009; Tsai et al., 2014; Okeke et al., 2016; Gong et al., 2024; Brundage et al., 1991). Furthermore, the observation that inhibition of SOCE but not IP3R affects cell migration in WT cells implies that the spatial pattern of the Ca^2+^ signal, rather than the specific molecular mediator, is key for effective cell migration.

This study highlights an important function of STIM proteins as key regulators of Ca^2+^ release through IP3Rs. Our findings indicate that when activated by ER Ca^2+^ depletion, STIM proteins facilitate a distinct form of diffuse Ca^2+^ release through IP3Rs. Multiple lines of evidence, both from the current study and from previous work, support this conclusion. First, we observe that global Ca^2+^ signals are diminished in cells lacking both STIM isoforms but are restored upon re-expression of either one of the two isoforms (Fig. 6). Second, consistent with previous studies indicating that the probability of IP3R channel opening and STIM activation are both regulated by similar levels of luminal ER Ca^2+^ (Vais et al., 2020; Stathopulos et al., 2008; Brandman et al., 2007), and that ER Ca^2+^ depletion shifts IP3R-mediated Ca^2+^ release from a localized to a more diffuse pattern (Lock and Parker, 2020), our analysis of IP3-mediated Ca^2+^ release reveals a significant transition from localized to global Ca^2+^ signals in cells expressing either STIM1 or STIM2, but not in cells deficient in both isoforms (Fig. 5). Importantly, this transition is not restored by the re-expression of the constitutively active STIM1 D76A mutant, which does not respond to ER Ca^2+^ depletion (Fig. 6). We further find that this shift is absent in most WT MDA-MB-231 cells, whereas in LM2-4 cells, a delayed onset of diffuse Ca^2+^ release occurs in approximately one third of the cells (Fig. S5 G). We suggest that the lower levels of expression and activity of STIM1 and STIM2 in MDA-MB-231 cells compared with LM2-4 cells (Fig. S5, D–F) or with the MDA-MB-231 STIM rescue cells underlie this observation. The lower resting ER Ca^2+^ levels in S1KO or S2KO cells compared with WT cells necessitate that the remaining STIM protein operates near its activation threshold. Therefore, in S1KO or S2KO cells but not in WT cells, a minor decrease in luminal ER Ca^2+^ leads to significant STIM activation. The initial Ca^2+^ puff activity following IP3 uncaging, which leads to a slight decrease in ER Ca^2+^ levels, would be sufficient to trigger a shift in the spatial pattern of IP3-mediated Ca^2+^ release in S1KO or S2KO cells, but not in WT cells.

The precise molecular mechanism through which STIM proteins affect IP3R-mediated Ca^2+^ release is presently unknown. Previous studies have reported inconsistent evidence regarding physical interaction between STIM and IP3Rs (Béliveau et al., 2014; Emrich et al., 2021; Sampieri et al., 2018; Ahmad et al., 2022; Woodard et al., 2010; Lur et al., 2009; Courjaret et al., 2025). Arguing against a strong physical association, pulldown experiments in HEK293 S1/S2 dKO cells heterologously expressing EYFP-STIM1 or EYFP-STIM1 D76A failed to detect such interaction (Fig. S5 H). Although the interaction between STIM and IP3R might be too weak and/or transient to be detected by co-IP, STIM could alternatively affect IP3R indirectly via an IP3R-interacting component. Potential candidates include proteins such as IRE1 that have been shown to physically associate with IP3R (Carreras-Sureda et al., 2019) and STIM1 (Carreras-Sureda et al., 2023), or annexin A1, which has been shown to play a critical role in the luminal ER Ca^2+^ regulation of IP3R (Vais et al., 2020; Wahl and Yule, 2020). While preparing this manuscript, work by Ivanova et al. introduced another intriguing alternative (Ivanova et al., 2024). The study presented evidence that binding to PI(4,5)P2 serves as a potent signal that sensitizes IP3Rs to IP3 and leads to a transition from localized to global Ca^2+^ release through IP3Rs. Based on this, it is possible that in breast cancer cells, STIM proteins may play a facilitating role in global Ca^2+^ release via IP3Rs by promoting interaction between IP3Rs and PI(4,5)P2 at ER-PM junctions. Given the intricate effects of IP3R-STIM crosstalk on cell migration, this proposed role of STIM aligns with recent findings, showing that gradients of ER-PM membrane contact sites play a crucial role in directing cell migration (Gong et al., 2024). Future studies of the effect of the type or morphology of ER-PM junctions on the pattern of IP3R Ca^2+^ release are required to address this possibility.

In summary, findings from this study reveal that particularly in breast cancer cells, STIM proteins are essential not only for SOCE but also for regulating the spatial characteristics of Ca^2+^ release through IP3Rs. This regulation plays a significant role in shaping cell migration.

## Materials and methods

### Cell culture and generation of knock-out cell lines

MDA-MB-231 and LM2-4 were cultured in Dulbecco’s modified Eagle’s medium (DMEM) supplemented with 10% fetal bovine serum (FBS), 1% penicillin/streptomycin (P/S), and 1% L-glutamine (L-Glu). MDA-MB-231 or LM2-4 cells were transduced with lentivirus expressing Cas9 and gRNAs. The infected cells were cultured in selection medium containing 1 μg/ml puromycin (for selection of STIM1 KO) or 1 μg/ml hygromycin (for selection of STIM2 KO). Then, the cells were seeded as a single cell into a 96-well plate for clone isolation. To obtain MDA-MB-231 or LM2-4 clones with deletion of both STIM isoforms, S2KO cells of either cell type were re-infected with lentivirus expressing Cas9 and STIM1 gRNAs and underwent clonal selection with puromycin. Successful deletion of either or both STIM isoforms was confirmed by WB analysis.

### Live-cell Ca^2+^ imaging

Before the day of the experiment, cells were cultured on fibronectin (10 mg/ml)-coated 18-mm glass coverslips or on Matrigel-coated polyacrylamide gels or 25-mm glass coverslips for 2 days. On the day of the experiment, cells were incubated at room temperature in Ringer’s solution (140 mM NaCl, 10 mM HEPES, 10 mM glucose, 0.8 mM MgCl_2_, 2.8 mM KCl, and 2 mM CaCl_2_, at pH 7.4) supplemented with 0.01% BSA and the indicated membrane-permeant fluorescent calcium indicator. Dye loading with Cal-520 AM or Cal-590 AM (5 μM; AAT Bioquest) was done for 1 h, and dye loading with Fura-2 AM (5 µg/ml; Invitrogen) was done for 30 min. For IP3 uncaging experiments, the membrane-permeant caged IP3 analog ci-IP3/PM (0.5 μM, #6210; Tocris) was also added to the dye and 0.01% BSA-supplemented Ringer’s solution. Following cell-loading procedure with membrane-permeant compounds, cells were incubated for 30 min in Ringer’s solution and then transferred onto an imaging chamber. Cytosolic Ca^2+^ levels were monitored by alternately exciting Fura-2–loaded cells at 340 and 380 nm, or by exciting Cal-520–loaded cells at 480 nm using a monochromator (Visitron), and collecting emitted light through a 515-nm long-pass filter. To record cytosolic Ca^2+^ levels in Cal-590–loaded cells, the cells were excited by illumination at 560 nm delivered by monochromator (Visitron) filtered by a 560/40-nm excitation filter and emission was collected with a 630/75-nm filter. Images were captured at room temperature every 2 or 3 s using either Olympus IX73 or IX83 microscopes equipped with either a cooled CCD camera (RETIGA R3, QImaging) or a back-illuminated sCMOS camera (Prime 95B; Photometrics), respectively, at 20× magnification (0.5 N.A. for IX73 or 0.75 N.A. for IX83). For each experiment, fluorescence traces from individual cells were recorded with VisiView (Visitron) or cellSens (Olympus) imaging software, and the average response was plotted using KaleidaGraph (Synergy).

### Analysis of ER Ca^2+^ content

48 h after infection with G-CEPIA_ER_–expressing lentiviruses, cells were seeded onto fibronectin-coated glass coverslips (10 µg/ml). Prior to imaging, glasses were placed in a recording chamber and cells were washed with Ca^2+^-free Ringer’s solution. Imaging of G-CEPIA_ER_ was conducted at room temperature by using Olympus IX83 microscopes. Imaging was performed with excitation at 488 nm and emission collected at 515 nm (long-pass filter) every 5 s. Fluorescence signals were detected with a back-illuminated sCMOS camera (Prime 95B; Photometrics) at 20× (0.75 N.A.) magnification. The fluorescence of G-CEPIA_ER_ was recorded for 1 min before and 10 min after treatment with Tg (0.4 µM) to deplete ER Ca^2+^. Fluorescence traces from individual cells were recorded offline with cellSens (Olympus) imaging software, and responses were plotted using KaleidaGraph (Synergy). To assess ER Ca^2+^ content under resting conditions, the basal G-CEPIA_ER_ fluorescence was divided by the fluorescence after Tg treatment for each cell.

### Analysis of Ca^2+^ puffs

Analysis of Ca^2+^ puffs was performed using a method adapted from Ellefsen et al. (2014). Following Ca^2+^ dye and ci-IP3/PM loading, as specified in the Live-cell Ca^2+^ imaging section, cells were incubated with the membrane-permeant calcium buffer EGTA/AM (5 μM; Invitrogen) for 45 min and subsequently washed with Ringer’s solution for an additional 30 min. The glass coverslip was placed in a recording chamber, and cells were incubated during the experiment in modified Ringer’s solution containing 70 μM Ca^2+^ to avoid Ca^2+^ permeation by SOCE. Total internal reflection fluorescence imaging was performed at room temperature using an Olympus IX83 microscope equipped with a cellTIRF-4Line system (405, 488, 561, and 640 nm lasers), a 100×/1.49 NA objective, and a back-illuminated sCMOS camera (Prime 95B; Teledyne Photometrics). 488-nm or 561-nm lasers were used to excite Cal-520 and Cal-590, respectively, and ci-IP3 was photoreleased using a 405-nm laser. Images of a 53.24 μm-by-44 μm field of view were acquired 10 s before and 60 s after ci-IP3 photolysis at 50 frames per second with 2 × 2 pixel binning (220 nm/pixel). The resulting VSI imaging files were converted to TIFF format using ImageJ/Fiji (NIH) and imported to FLIKA for processing. From the 500 initial frames before ci-IP3 photolysis, 300 frames (∼6 s) were averaged to produce a ratio image stack (F/F0). The stack was Gaussian-filtered, and pixels above a threshold of 1.0 were identified. The “Detect-puffs” plug-in was used to quantify puff sites, events, amplitudes, and durations of localized Ca^2+^ signals. Results were saved in Excel (Microsoft), and processed summary panels were prepared with KaleidaGraph (Synergy).

### Colony formation assay

The soft agar culturing setting comprised bottom and upper layers. To prepare the bottom layer, agarose was combined with DMEM (supplemented with 10% FBS, 1% P/S, and 1% L-Glu) to achieve a final concentration of 0.5% (wt/vol). The mixture was heated until all agarose aggregates were fully dissolved, using gentle heating to prevent bubble formation. A 3 ml portion of the dissolved agarose was added to each well of a 6-well plate and incubated at 4°C for 1 h to solidify. Then, to prepare the upper layer, agarose was mixed with DMEM (supplemented with 10% FBS, 1% P/S, 1% L-Glu) to a final concentration of 0.3% (wt/vol) and with or without 10 μg/liter fibronectin and heated until completely dissolved. The solution was then cooled to around 37°C that is compatible with cell viability. After cooling, the agarose solution was mixed with cells (5,000 cells/well) and placed onto the solidified bottom layer. The plate was incubated at 37°C in a 5% CO_2_ environment for 3 wk, and fresh growth medium was carefully added every 2–3 days. After 3 wk, the agar-containing plates were fixed with 4% paraformaldehyde (PFA) for 20 min, stained with 0.05% crystal violet for 10 min, and washed several times with distilled water (DDW). The agar culturing surface was imaged using an inverted microscope (DMi8; Leica) equipped with a Leica DFC7000 T camera at 10× (N.A 0.22) magnification, and images were analyzed with ImageJ/Fiji (NIH) software to quantify the number of colonies in each well.

### Analysis of cell proliferation

Cells were seeded in a 96-well plate (5,000 cells/well) and cultured in DMEM supplemented with 10% FBS, 1% P/S, 1% L-Glu, and 10% vol/vol alamarBlue (Thermo Fisher Scientific) for 96 h. Cell viability was monitored every day by measuring absorbance at 570 and 600 nm using a plate reader (Tecan).

### Tumor formation assay

The MDA-MB-231 cells were cultured in a 10-cm^2^ plate at 80% of confluence. Cells were harvested with 0.25% trypsin and centrifuged at 400 *g* for 5 min. Cells were then counted and suspended in serum-free media. 1.5 × 10^6^ cells/50 μl were injected into the mammary fat pad of 6- to 8-wk-old NOD/SCID female mice weighing 18–21gr, and tumor growth was monitored for 36 days. Tumor volume was measured using a digital caliper and calculated according to the formula:

$$V=\frac{L\times W^{2}}{2}$$

where

(1) L (length) is the longest side of tumor,
(2) W (width) is the shortest side of tumor,
(3) A common assumption is W = H (height),

Mice were sacrificed when tumor size reached 1,500 mm^3^. The experimental protocol was approved by the Animal Care and Use Committee of the Technion (Haifa, Israel).

### Generation of vectors and virus preparation

To generate vectors expressing Cas-9 and gRNAs for STIM1 or STIM2, two different STIM1 gRNA sequences were cloned into a LentiCRISPRv2 vector (#52961; Addgene) and a single STIM2 gRNA sequence was cloned into a LentiCRISPRv2 vector (#91977; Addgene). The STIM1 gRNA sequence used in MDA-MB-231 cells was 5′-GTA​TGC​GTC​CGT​CTT​GCC​CTG-3′, and the STIM1 gRNA sequence used in LM2-4 cells was 5′-TGA​GGA​TAA​GCT​CAT​CAG​CG-3′. The STIM2 gRNA sequence, used in either MDA-MB-231 or LM2-4 cells, was 5′-CAT​GAG​CGC​CGG​GCT​ATC​G-3′. To generate Lenti-hSTIM1-YFP, Lenti-hSTIM1-D76A-YFP, and Lenti-hSTIM2-YFP, the coding region from the previously reported (Zomot et al., 2021) YFP-STIM1, YFP-STIM2, and YFP-STIM1 D76A vectors was cloned into a NSPI-CMV MCS lentiviral expression vector (a generous gift from Aaron Ciechanover’s laboratory, Technion - Israel Institute of Technology, Haifa, Israel). All plasmids were sequenced at the Technion Biomedical Core Facility for verification.

To prepare the different lentiviruses, HEK293T cells were co-transfected with the packaging vector ps-PAX2, the envelope vector pMD2G, and either one of the following transfer vectors: G-CEPIA1er (#164590; Addgene), Lenti-hSTIM1-YFP, Lenti-hSTIM1-D76A-YFP, Lenti-hSTIM2-YFP, LentiCRISPRv2 vector (#52961; Addgene) harboring STIM1 gRNA sequences, or LentiCRISPRv2 vector (#91977; Addgene) harboring STIM2 gRNA sequence. The supernatant containing the lentivirus was collected 72 h after transfection and filtered through a 0.45-μm syringe filter.

### Immunocytochemistry

Cells were seeded onto fibronectin-coated glass coverslips (10 µg/ml) and cultured in DMEM at 37°C. For paxillin staining, cells were cultured for 4 h, whereas for phosphorylated MLC staining, cells were cultured overnight. After incubation, cells were washed with PBS and fixed for 10 min in PBS containing 4% PFA and 0.2% Triton X-100. Permeabilization was performed for 10 min using PBS with 0.1% Tween and 0.1% Triton X-100. Cells were then blocked for 20 min in PBS containing 0.1% Tween, 0.1% Triton X-100, and 1% BSA. All subsequent washing steps were carried out using the blocking solution. Co-staining with phalloidin (Phalloidin-iFluor 488; Abcam) was performed in all cases. Primary antibody incubation occurred overnight at 4°C, followed by washes and secondary antibody incubation. Following staining, slides were stored at −20°C until imaging. Images were captured using a Zeiss LSM 880 confocal microscope with a 63× (1.40 NA) objective and 488- and 561-nm lasers. Emission from the 488-nm laser was collected between 505 and 580 nm (green channel), while emission from the 561-nm laser was collected between 583 and 685 nm (red channel). Image processing was performed using Zen Airyscan processing, and quantification of secondary antibody staining was performed using ImageJ/Fiji (NIH) software. To quantify the relative intensity of pMLC at cortical regions, confocal images were first thresholded based on the phalloidin channel. The mean pMLC intensity within this masked region was then measured and normalized to the mean pMLC intensity across the entire cell. To quantify the number of focal adhesions, raw images of paxillin intensities were processed in ImageJ/Fiji (NIH) using the following workflow. Background was subtracted using the sliding paraboloid method with a rolling ball radius of 10 pixels. A gamma correction with a value of 2 was applied, followed by median filtering with a five-pixel radius to reduce noise. Brightness and contrast were automatically adjusted, and images were thresholded using the autothreshold function. The resulting images were converted to binary, and particle analysis was performed using the “Analyze Particles” tool to obtain the area and number of focal adhesions per cell. The number of focal adhesions was normalized to the total cell area.

### Western blotting

The cells were seeded onto a 60-mm^2^ plate at high (∼80–90%) confluence and cultured overnight. Then, the cells were washed three times with cooled PBS and collected into cooled lysis buffer (50 mM Tris-Cl, pH 7.6, 150 mM NaCl, 50 mM EDTA, 1% of IGEPAL, and a cocktail of protease inhibitors [cOmplete ULTRA tablets; Roche]) on ice. The cell lysates were incubated for 15 min on ice and centrifuged for 10 min (16,000 *g*) at 4°C. Then, a small sample of the lysates was used for determination of the total protein concentration and the appropriate volume of lysate was mixed with 4X sample buffer solution and heated for 10 min at 95°C. The processed lysates were loaded into SDS–polyacrylamide gel (7.5 or 10% acrylamide) electrophoresis and run in TG-SDS buffer. After in-gel protein separation, the proteins were transferred to nitrocellulose membrane (Bio-Rad). The membrane was washed with TBS-T, blocked with 5% fat milk in TBS-T, and exposed overnight to a primary antibody at 4°C. Following this step, the membrane was washed three times and incubated with an HRP-conjugated secondary antibody, diluted in 1% fat milk (1:10,000, 1 h, room temperature). After another washing step, luminescence was analyzed using an enhanced chemiluminescence (ECL) detection system (Mercury or Bio-Rad). WB quantification was carried out using the “Analyze Gels” tool in ImageJ/Fiji (NIH). The following primary antibodies were used: STIM1 (1:1,000, ACC-063; Alomone), STIM2 (1:1,000, ACC-064; Alomone), Orai1 (1:1,000, sc-377281; Santa Cruz Biotechnology), IP3R-1 (1:1,000, ACC-019; Alomone), IP3R-2 (1:500, sc-398434; Santa Cruz Biotechnology), IP3R-3 (1:2,000, BD 610312; BD Transduction Laboratories), pMLC (1:1,000 ab2480; Abcam or 1:500 cs3675; Cell Signaling), vimentin (1:1,000, sc-7557-R; Santa Cruz Biotechnology), Snail (1:1,000, AP2054a; Abgent), E-cadherin (1:2,000, BD 610182; BD Transduction Laboratories), tubulin (1:5,000, 137585; Sigma-Aldrich), GAPDH (1:10,000, ab8245; Abcam), and actin (1:1,000, ms1295 P0; Sigma-Aldrich). The horseradish peroxidase–conjugated secondary antibodies used were from Jackson ImmunoResearch (705-036-147, 115-036-003, 111-036-003).

### RNA interference

The esiRNA constructs against human ITPR1 (EHU094011), ITPR2 (EHU056071), ITPR3 (EHU118761), or siControl (Mission siRNA Universal Negative Control #2) were purchased from Merck. Cells were transfected with a mixture of Lipofectamine RNAiMAX (Invitrogen) and either ITPR1 esiRNA, ITPR2 esiRNA, ITPR3 esiRNA, or a 1:1:1 mix of all three esiRNAs. Following transfection, cells were incubated for 48 h prior to usage.

### Co-immunoprecipitation

24 h following transfection, HEK293 S1/S2 dKO cells (Emrich et al., 2021) were washed with PBS solution and placed into lysis buffer (150 mM NaCl, 25 mM Tris-Cl, pH 7.6, 10% glycerol, 1 mM EDTA, 0.5% Triton X-100) containing protease inhibitors (Roche) on ice for 30 min. Samples were homogenized by pipetting and centrifuged for 20 min at 17,000 *g*. IP was performed using magnetic bead–conjugated anti-GFP beads (ChromoTek, GFP-Trap) by incubation in the lysis buffer for 1 h on ice with rocking. The beads were washed three times in lysis buffer before elution into sample buffer. Following elution, SDS-PAGE, WB, and ECL analyses were performed as detailed above. Primary antibodies used were for IP3R-2 (1:500, sc-398434; Santa Cruz Biotechnology), IP3R-3 (1:2,000, BD 610312; BD Transduction Laboratories), or STIM1 (1:1,000, ACC-063; Alomone).

### Transwell Boyden chamber assay

Cell migration was evaluated using a 24-well Boyden chamber (Falcon) with an 8-µm pore insert membrane coated with fibronectin (10 µg/ml; Invitrogen). For invasion assays, the membrane was additionally coated with Matrigel (Corning). The chambers were first rehydrated by adding serum-free culture medium. The medium was then replaced with a serum-free cell suspension containing 2 × 10^5^ cells/in 200 μl. The lower chamber was filled with 750 μl of culture medium containing 10% FBS as a chemoattractant. Cells were incubated for 16 h at 37°C and 5% CO_2_. In experiments shown in Fig. 3, the following compounds were added to cells: 2-APB (Tocris), nifedipine (Alomone), SKF96365 (Alomone), Xes-C (Tocris), NS8593 (Alomone), and ML-9 (Tocris). After incubation, cells were fixed (4% PFA), permeabilized, and stained with 0.5% crystal violet. For each insert, five to seven representative images were captured by an inverted microscope (Leica DMi8) equipped with a Leica DFC7000 T camera at 10× (N.A. 0.22) magnification and the relative number of migrating cells was quantified with ImageJ/Fiji (NIH).

### Cell adherence assay

Cells were harvested using 0.25% trypsin, resuspended in DMEM supplemented with 10% FBS, 1% P/S, and 1% L-Glu, and subsequently seeded onto fibronectin-coated 96-well plates. After 30 min, the cells were washed with PBS to eliminate nonadherent cells. The nuclei of the remaining adherent cells were fixated with cold methanol and stained with DAPI, and images of the bottom of each well were acquired with an Olympus IX83 microscope equipped with a back-illuminated sCMOS camera (Prime 95B; Teledyne Photometrics) at 4× magnification (NA 0.13). The number of cells was calculated from each image using ImageJ/Fiji (NIH).

### Measurement of total F-actin

Estimation of total F-actin was performed using a method adapted from Condeelis and Hall (1991). Cells were cultured overnight in fibronectin-coated 96-well plates. Cells were then simultaneously fixed, permeabilized, and stained with Phalloidin-iFluor 488 (Abcam) using either buffer A (4% formaldehyde, 0.2% Triton X-100, 20 mM KPO_4_, 10 mM HEPES, 5 mM EGTA, 2 mM MgCl_2_, 0.1% saponin, pH 6.8) or buffer B (PBS supplemented with 0.2% Triton X-100 and 4% formaldehyde) for 20 min at room temperature. Following this procedure, the bottom of each well was imaged and the number of cells per well was quantified. Cells were then washed with saponin buffer (0.1% saponin, 20 mM KPO_4_, 10 mM HEPES, 5 mM EGTA, 2 mM MgCl_2_, pH 6.8) and incubated with 100% methanol for 30 min to release the total bound phalloidin. The total amount of Phalloidin-iFluor 488 released was measured using a plate reader (Cytation 5, excitation: 480 nm; emission: 530 nm) and normalized to the total number of cells per well.

### Preparation of polyacrylamide gels of varying stiffness

Glass-bottom plates (30 mm; Greiner) were first activated using a 2% solution of 3-aminopropyltrimethoxysilane followed by 1% glutaraldehyde. PA/Bis-acrylamide solutions with varying concentrations, 12/0.28% (shear modulus ∼30,067 Pa), 7.5/0.03% (∼1,535 Pa), and 3/0.06% (∼230 Pa), were prepared by mixing with 0.02% carboxylated red FluoSpheres (Life Technologies), 0.05% ammonium persulfate, and 0.4% TEMED (Bio-Rad). For each activated plate, 7.5 μl of the PA mixture was applied and overlaid with a glass coverslip coated with Matrigel, which transferred to the PA surface during polymerization. Gels were allowed to polymerize at room temperature for 30 min. Afterward, the PA substrates were incubated in PBS for at least 1 h, and the coverslips were carefully removed using a razor blade (Aratyn-Schaus et al., 2010).

### Statistical analysis

Comparison of two groups was performed with standard Student’s *t* test (two-tailed, unequal variance) and comparison of more than two groups with ANOVA with Tukey’s post hoc test. Data distribution was assumed to be normal, but this was not formally tested.

### Online supplemental material

Fig. S1 provides additional data on STIM1 and STIM2 expression in MDA-MB-231 and LM2-4 cells following CRISPR/Cas9 gene editing. The figure also shows results from adhesion and invasion assays using WT, S1KO, S2KO, and dKO MDA-MB-231 cells, as well as STIM1 and STIM2 re-expression in MDA-MB-231 dKO cells. Fig. S2 shows WB analysis of EMT markers and NFAT1 expression in MDA-MB-231 and MCF-7 cells, along with F-actin as a loading control. Fig. S3 includes data on IP3R expression and function in MDA-MB-231 and LM2-4 cells. The data highlight the role of IP3Rs in cell migration under altered Ca^2+^ conditions and following siRNA knockdown. Fig. S4 shows Ca^2+^ signaling responses in cells cultured on substrates of varying stiffness and provides kinetic analysis of Ca^2+^ puffs in WT, S1KO, S2KO, and dKO cells of both MDA-MB-231 and LM2-4 cell types, as well as STIM1 and STIM2 rescue in MDA-MB-231 dKO cells. The figure also shows summary of resting Ca^2+^ measurements using IP3 uncaging conditions. Fig. S5 contains detailed analyses of STIM1 and STIM2 expression and SOCE responses in LM2-4 cells, including comparisons with MDA-MB-231 cells and immunoprecipitation data showing lack of interaction between STIM1 and IP3R2 or IP3R3. Videos 1, 2, 3, 4, 5, 6, 7, 8, 9, 10, and 11 show TIRFM time-lapse imaging of Ca^2+^ dynamics in WT, S1KO, S2KO, and dKO of MDA-MB-231 and LM2-4 cells, as well as in MDA-MB-231 dKO cells expressing EYFP-STIM1, EYFP-STIM1 D76A, or EYFP-STIM2 following UV-induced IP3 uncaging.

## Supplementary Material

SourceData F2 is the source file for Fig. 2.

SourceData F3 is the source file for Fig. 3.

SourceData FS1 is the source file for Fig. S1.

SourceData FS2 is the source file for Fig. S2.

SourceData FS3 is the source file for Fig. S3.

SourceData FS5 is the source file for Fig. S5.

## Data Availability

All data supporting the findings of this study are included in the main text and supplementary information files. Additional information is available from the corresponding author upon reasonable request.
