# Supplementary material for: STIM-IP3R crosstalk regulates migration of breast cancer cells
Source: J Cell Biol. 2025 Jul 28;224(9):e202411203. doi: 10.1083/jcb.202411203 (PMC12302952; doi:10.1083/jcb.202411203)
Supplement: SourceData F2 — is the source file for Fig. 2. [file jcb_202411203_sourcedataf2.pdf]

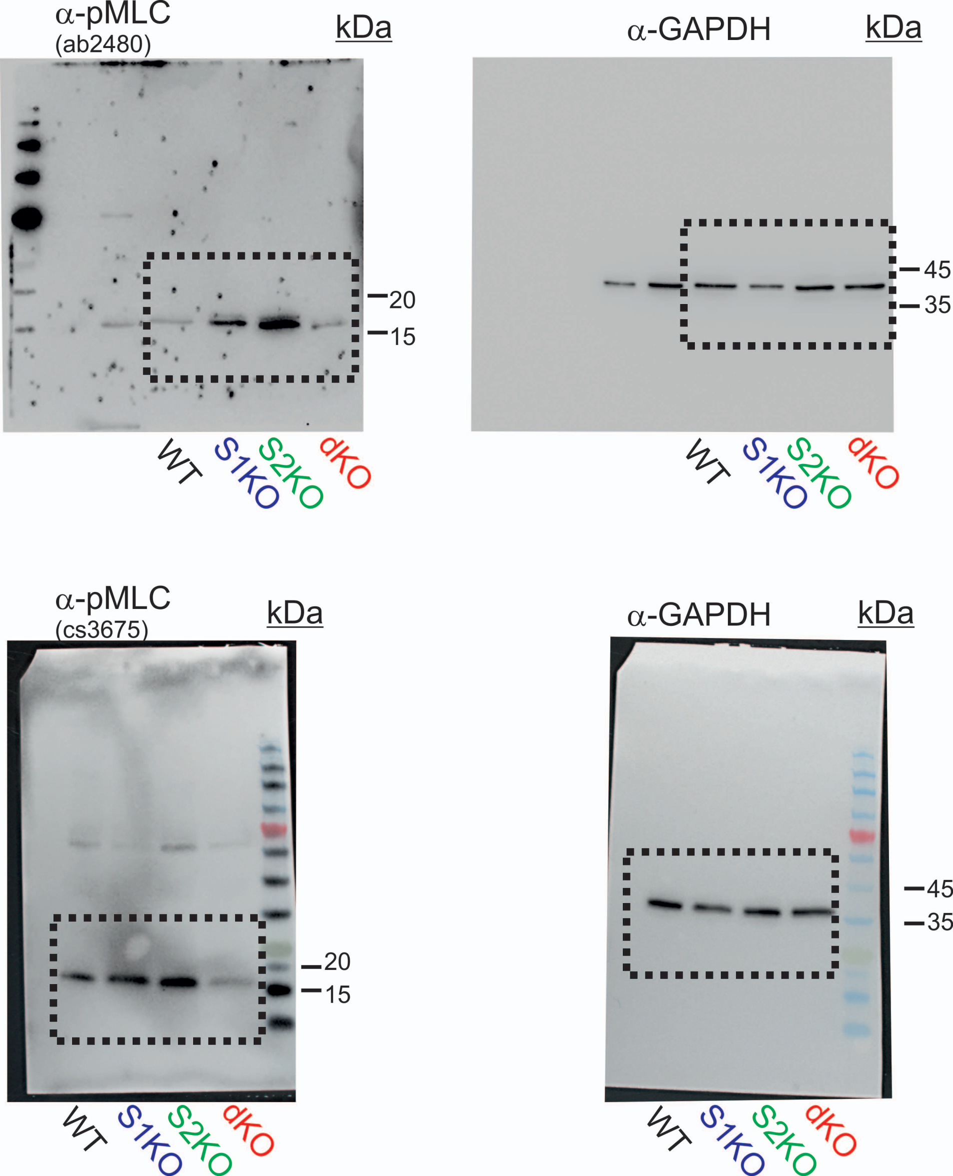
α-pMLC
(ab2480)
kDa
20
15
WT
S1KO
S2KO
dKO
α-GAPDH
kDa
45
35
WT
S1KO
S2KO
dKO
α-pMLC
(cs3675)
kDa
20
15
WT
S1KO
S2KO
dKO
α-GAPDH
kDa
45
35
WT
S1KO
S2KO
dKO
