# Supplementary figures and images for: STIM-IP3R crosstalk regulates migration of breast cancer cells
Source: J Cell Biol. 2025 Jul 28;224(9):e202411203. doi: 10.1083/jcb.202411203 (PMC12302952; doi:10.1083/jcb.202411203)

siRNA:

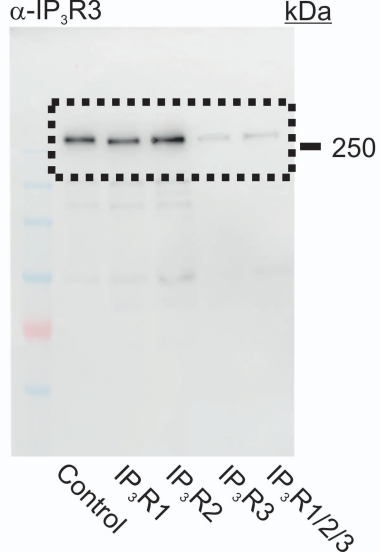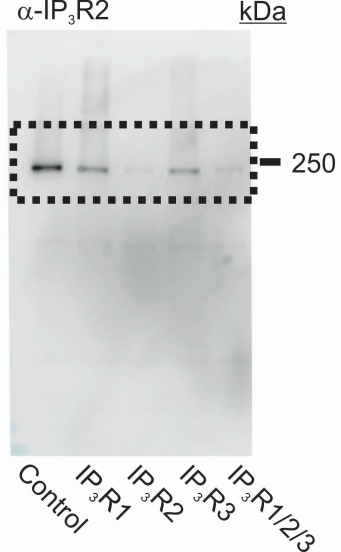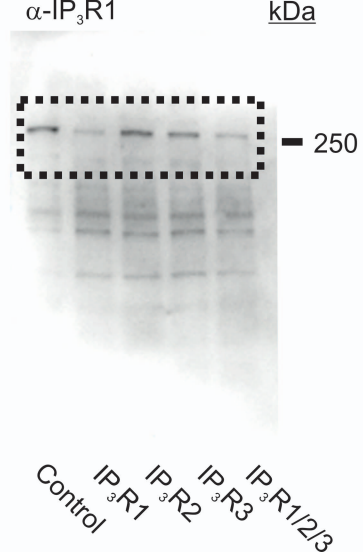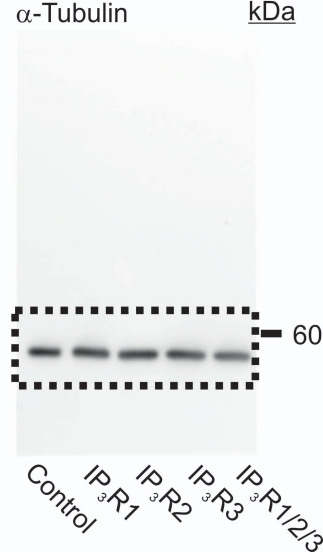

Supplement: SourceData F3 — is the source file for Fig. 3. [file jcb_202411203_sourcedataf3.pdf]

# A

## MDA-MB-231

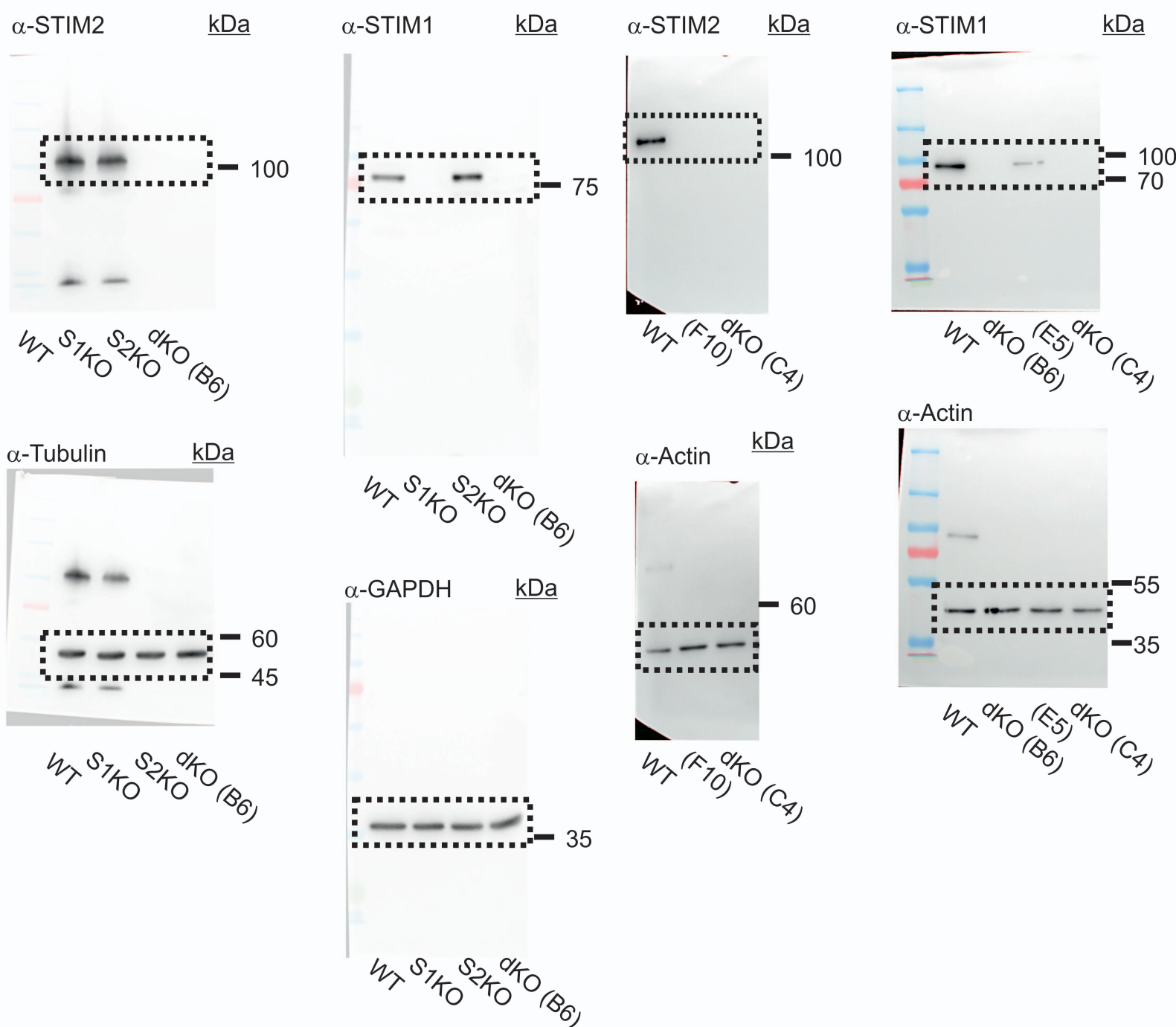

# B

## LM2-4

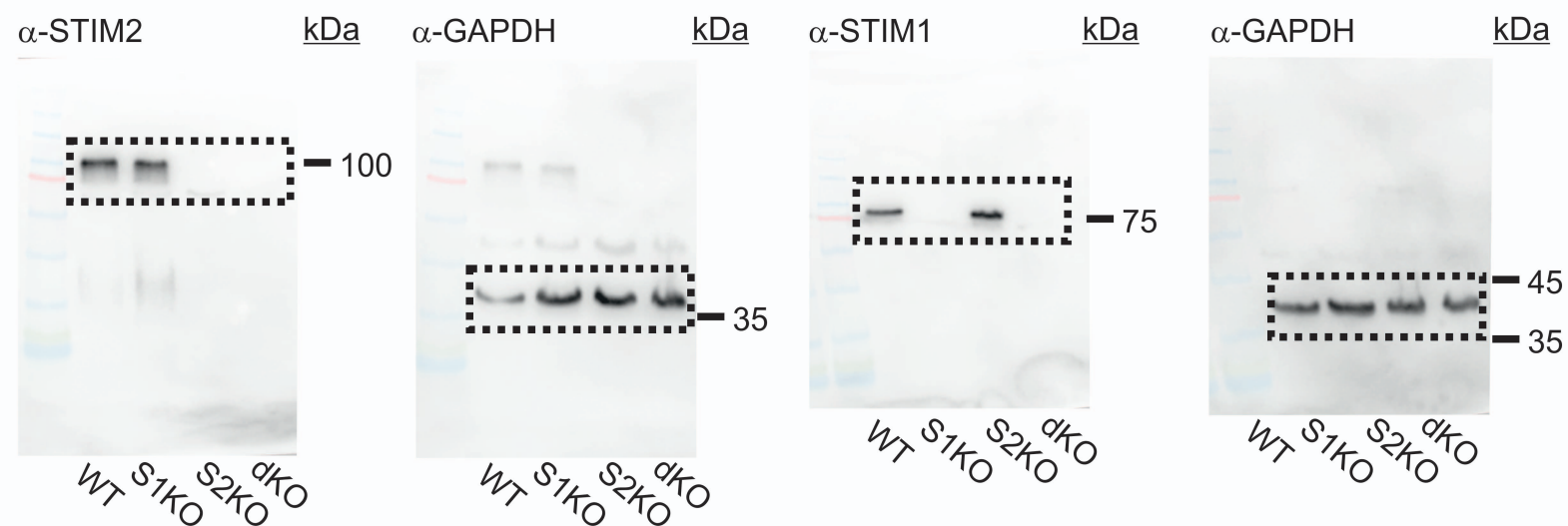

Supplement: SourceData FS1 — is the source file for Fig. S1. [file jcb_202411203_sourcedatafs1.pdf]

## MDA-231

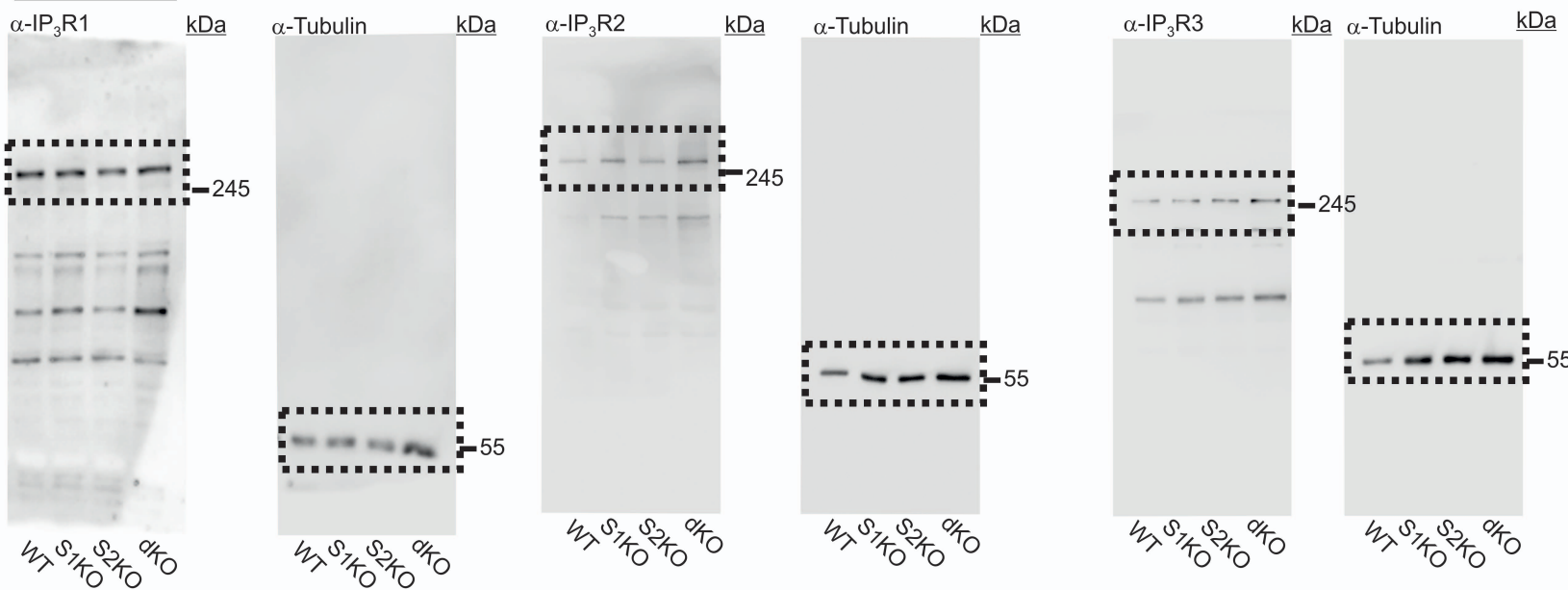

## LM-2-4

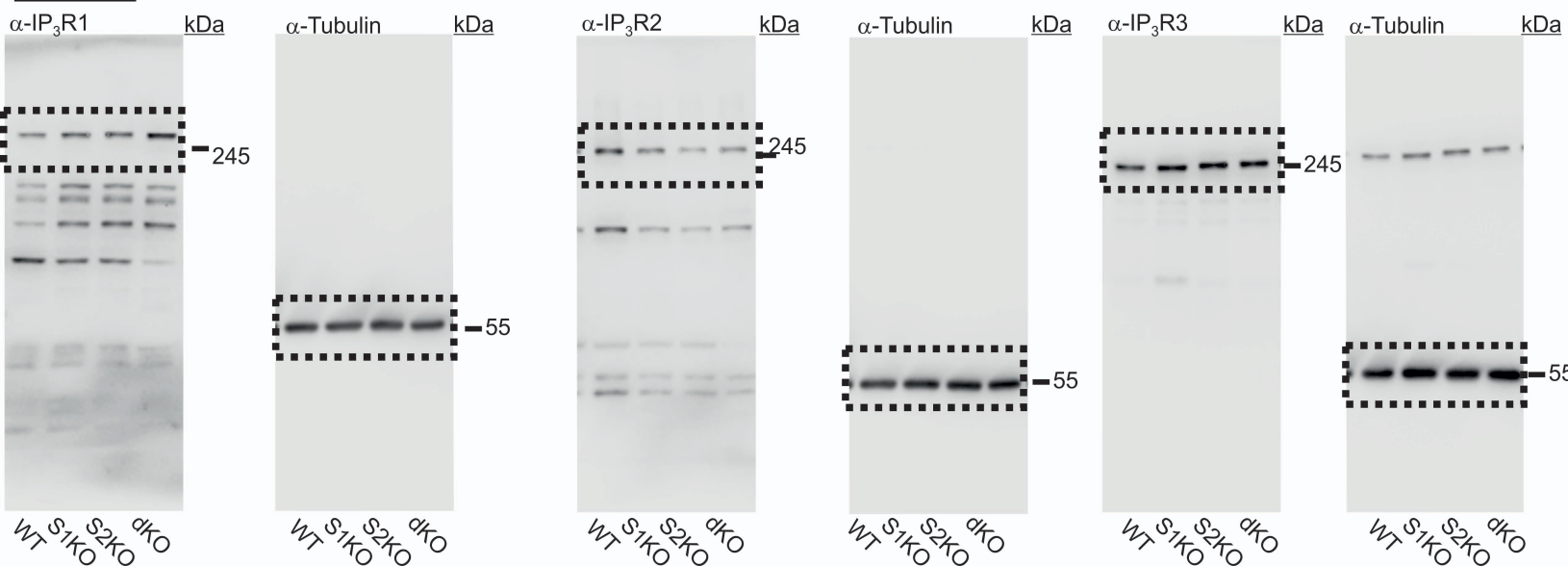

Supplement: SourceData FS3 — is the source file for Fig. S3. [file jcb_202411203_sourcedatafs3.pdf]

E

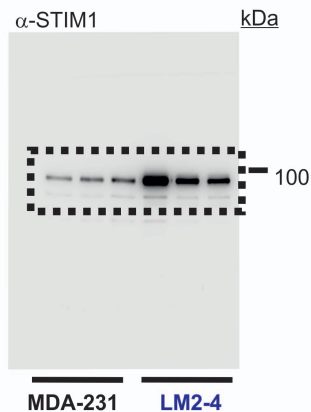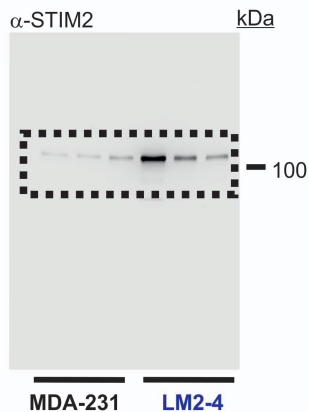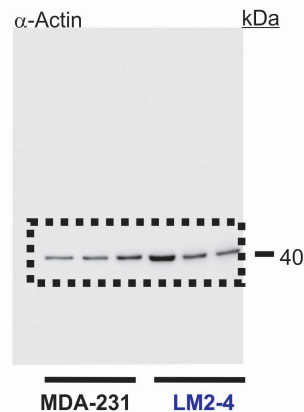

H

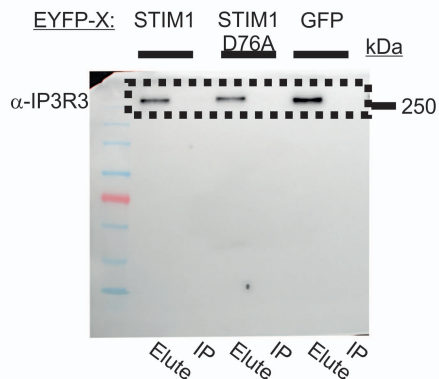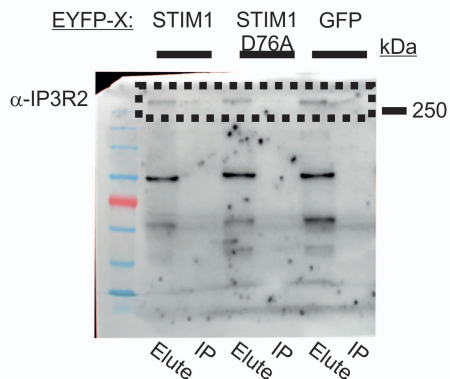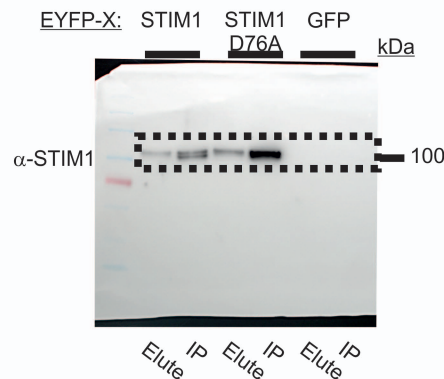

Supplement: SourceData FS5 — is the source file for Fig. S5. [file jcb_202411203_sourcedatafs5.pdf]
