# Supplementary material for: STIM-IP3R crosstalk regulates migration of breast cancer cells
Source: J Cell Biol. 2025 Jul 28;224(9):e202411203. doi: 10.1083/jcb.202411203 (PMC12302952; doi:10.1083/jcb.202411203)
Supplement: SourceData FS2 — is the source file for Fig. S2. [file jcb_202411203_sourcedatafs2.pdf]

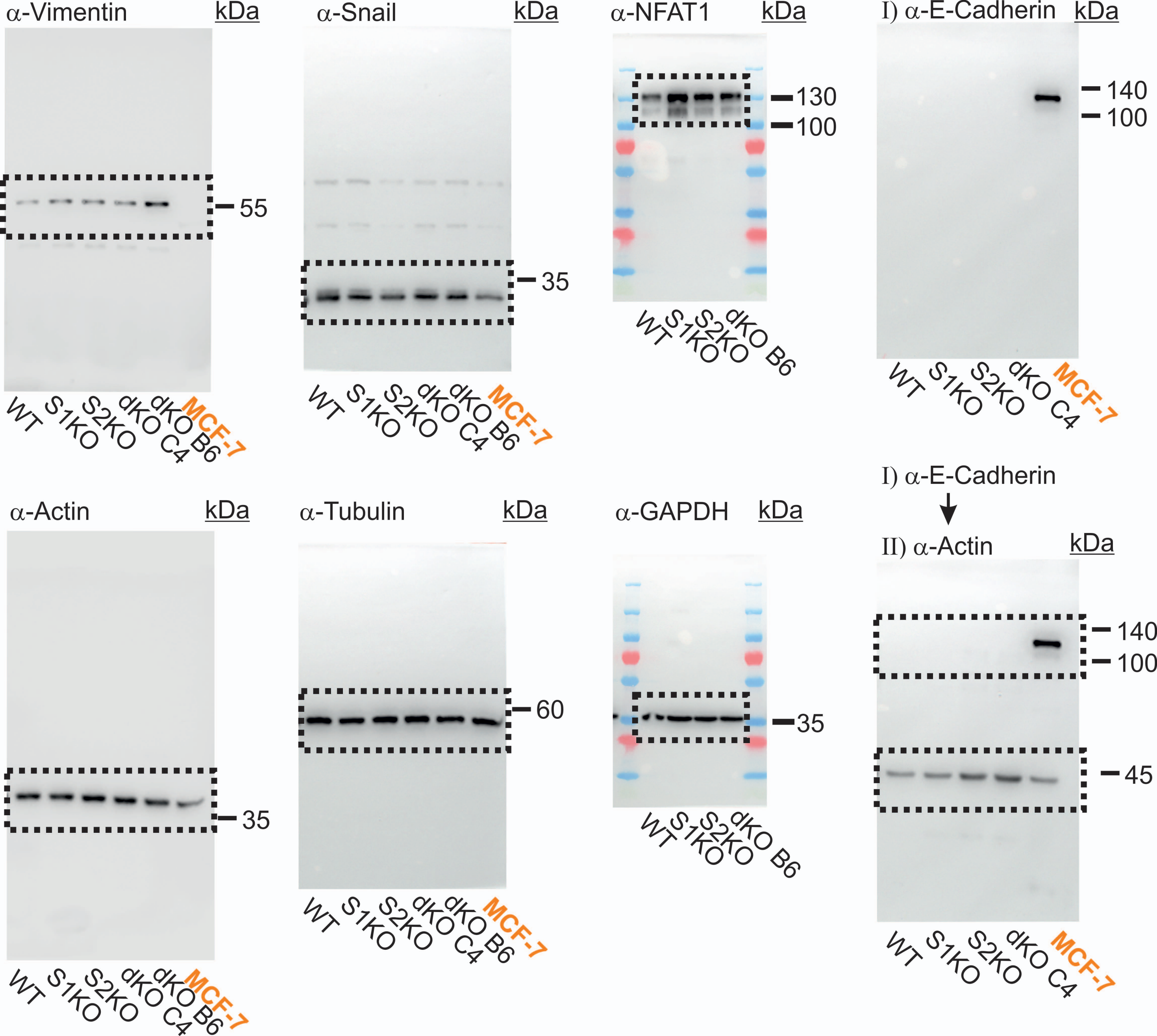

α-Vimentin
kDa
55
WT
S1KO
S2KO
dKO C4
dKO B6
MCF-7
α-Snail
kDa
35
WT
S1KO
S2KO
dKO C4
dKO B6
MCF-7
α-NFAT1
kDa
130
100
WT
S1KO
S2KO
dKO B6
I) α-E-Cadherin
kDa
140
100
WT
S1KO
S2KO
dKO C4
MCF-7
α-Actin
kDa
35
WT
S1KO
S2KO
dKO C4
dKO B6
MCF-7
α-Tubulin
kDa
60
WT
S1KO
S2KO
dKO C4
dKO B6
MCF-7
α-GAPDH
kDa
35
WT
S1KO
S2KO
dKO B6
I) α-E-Cadherin
II) α-Actin
kDa
140
100
45
WT
S1KO
S2KO
dKO C4
MCF-7
